# Biological therapy: approaches in colorectal cancer. Strategies to enhance carcinoembryonic antigen (CEA) as an immunogenic target.

**DOI:** 10.1038/bjc.1998.114

**Published:** 1998-03

**Authors:** A. P. Zbar, N. R. Lemoine, M. Wadhwa, H. Thomas, D. Snary, W. A. Kmiot

**Affiliations:** Academic Department of Colorectal Surgery, Hammersmith Hospital, London, UK.


British Journal of Cancer (1998) 77(5), 683–693
© 1998 Cancer Research Campaign

Review

### Biological therapy: approaches in colorectal cancer

### Strategies to enhance carcinoembryonic antigen (CEA) as an immunogenic target

**AP Zbar,[1] NR Lemoine,[2] M Wadhwa,[3] H Thomas,[4] D Snary[5] and WA Kmiot[1]**

[1]Academic Department of Colorectal Surgery, Hammersmith Hospital, London, UK; [2]Molecular Pathology Laboratory, Imperial Cancer Research Fund, Imperial College School of Medicine, Hammersmtih Campus, London, UK; [3]National Institute for Biological Standards and Control, Hertfordshire, UK; [4]Department of Clinical Oncology, Imperial College School of Medicine, Hammersmtih Campus, London, UK; [5]Applied Development Laboratories, Imperial Cancer Research Fund

In the UK, almost 20 000 people die each year from colorectal cancer. Despite a potential curability rate of 70% or greater, the overall survival at 5 years is just over 30%; a figure that has changed little over the last 4 decades despite advances in adjuvant and therapeutic chemotherapy and radiotherapy (King's Fund Forum, 1990).

Moreover, there is evidence that over half the patients operated upon for cure have occult metastatic disease at the time of initial surgery (August et al, 1994). Recent novel immunocytochemical techniques using immunobead polymerase chain reaction (PCR) for detection of the tumour-associated antigen carcinoembryonic antigen (CEA) have permitted the identification of single malignant cells in peripheral blood samples, bone marrow aspirates and peripheral stem cell harvests through the recognition of unique hybrid gene transcripts. (Schlimok et al, 1990; Lindeman et al, 1992; Hardingham et al, 1993; Johnson et al, 1995).

Although the presence of such cells in the bone marrow at the time of preliminary colon resection appears to be associated with a worse prognosis (Riethmuller and Johnson, 1992) the number of cells that correlates with a poor outcome is unknown, and the relationship between the development of secondary disease and circulating tumour cells remains poorly understood (Osborne et al, 1991). Phage cloning and hybridization have taken advantage of the limited but specific genetic alterations in developing large bowel neoplasms to detect *ras* mutations in colorectal cancer cells in the stool (Sidransky et al, 1992). The demonstration of small tumour burdens of this type in which cells are exposed in unshielded mesenchymal locations may provide relatively novel immunotherapeutic and chemoimmunotherapeutic targets and identify surrogate end points in treatment that may prove superior to crude survival time.

Immunotherapeutic strategies in advanced colorectal cancer have generally met with little success. Conventional treatments have largely relied on either interleukin 2 (IL-2) or adoptive IL-2-stimulated tumour infiltrating lymphocytes (TILs), with only sporadic reports of tumour regression (Rosenberg et al, 1989; Kradin et al, 1989). Recently, there has been a resurgence of interest in immunotherapy (and the potential of gene therapy) in advanced colorectal cancer and in an adjuvant setting. The adjuvant use of the murine monoclonal $IgG_{2a}$ antibody, 17-1A directed against the CO 17-1A surface epitope of CEA (found in up to 80% of colorectal carcinomas) has resulted in an improvement in disease-free survival and a reduction in locoregional recurrence rates of almost 30% in patients with Dukes' C carcinoma compared with untreated controls (Riethmuller et al, 1994). Carcinoembryonic antigen (CEA), a surface-expressed tumour-associated antigen, is a well-characterized glycoprotein represented in high density on most malignant tumours of the gastrointestinal tract (Muraro et al, 1985). The immunogenicity of CEA as a potential target antigen in colorectal cancer is at present unclear, with variable reports of inducible humoral and cell-mediated responsiveness to CEA epitopes in patients with different stages of disease. There is much that remains unknown regarding the natural immunological response to a native antigen such as CEA both in terms of its antigenic processing and its potentially immunodominant epitopes.

This review assesses the role of CEA as a 'natural' autoantigen along with strategies that render epitopes of CEA potentially immunogenic. This may be achieved by the use of xenogeneic, chimaeric, humanized or wholly human monoclonal and polyclonal antibodies and with anti-idiotypic therapy. The advantages and limitations of each strategy and their potential role in the treatment of advanced colorectal cancer are discussed.

*Received 11 June 1997*
*Revised 9 September 1997*
*Accepted 12 September 1997*

*Correspondence to:* AP Zbar, Surgical Directorate, Hammersmith Hospital, DuCane Road, London W12 0HS, UK

### MECHANISMS OF TUMOUR ESCAPE FROM IMMUNOLOGICAL RECOGNITION

The variability of tumours permits their escape from immune recognition. An improvement in the understanding of the immunobiology of cell-mediated anti-tumour defences as well as a better knowledge of tumour recognition molecules expressed on the surface of many tumours has permitted the development of new anti-tumour strategies to stand alongside conventional chemotherapy and radiotherapy in colorectal cancer.

Isolated tumour cells are able to be eliminated by several conventional immunological mechanisms, most notably antibody dependent cellular cytotoxicity (ADCC) (Steplewski et al, 1983; Adams et al, 1984) complement-dependent cytolysis (Herlyn and Koprowski, 1981) and apoptosis (Trauth et al, 1989). Knowledge of cell surface regulatory molecules expressed on tumour cells may enhance natural apoptosis and tumour regression (Wyllie et al, 1980).

**Figure 1** Mechanism of MHC molecule antigen processing. (**A**) Class 1 MHC antigen processing. Proteasomes digest cytoplasmic protein into processed peptides (eight or nine amino acids in length). These peptides adhere to the endoplasmic reticulum by polymorphic transporter proteins (Tap-1 and Tap-2). Chaperonin molecules detain empty MHC class I molecules in the endoplasmic reticulum for association with processed antigen and transfer to the cell surface. (**B**) Class II MHC antigen processing. Foreign antigen is endocytosed and after processing (peptides 15–25 amino acids in length), the peptide is aggregated in the Golgi apparatus with α, β and γ components of the MHC class II molecule formed in the endoplasmic reticulum. After complexing with foreign peptide, the γ-chain is degraded and the αβ heterodimer/processed antigen is expressed on the cell surface for Th TcR recognition. The mechanism of transport of the complex from the Golgi apparatus to the cell membrane is unknown

**Figure 2** Mechanism of T-cell receptor (αβ) complex interaction with antigen presenting cell. T cells have a dual specificity for MHC molecules and processed antigen. The TcR αβ is complexed with CD3, which has an intracytoplasmic component for signal transduction after occupancy with processed antigen. The CD4 molecule secondarily interacts with MHC class II to produce local cytokines (IL-2 and IFN-γ) for Th cell support and transformation of B cells. CD 28 initiates signal transduction independently to augment local cytokine production using B7 as a ligand molecule

The finding of unique tumour-associated antigens (TAAs) in solid malignancies has presented a range of important targets for adoptive humoral and cellular immune therapies. Early attempts to define TAAs in murine models by immunizing mice against either spontaneous tumours or chemically and virally induced tumours were confused by general species reactivity to normal transplantation antigens. The development of syngeneic mice with identical histocompatibility antigen expression systems permitted cutaneous but not tumour transplantation, implying the presence of tumour-specific antigenicity. Most work on tumour immunology has centred on cytotoxic T-cell (CTL) activity since the demonstration that the ability to reject tumours can be adoptively transferred by lymphocytes and not by serum.

The recognition of foreign tumour antigens requires a presentation to immunocytes in the form of target fragments linked to the major histocompatibility complex (MHC) class I and class II molecules interacting with the T-cell receptor (TcR) mechanism. Intracellular antigens, such as oncogene products and extracellular (or foreign) antigens, are handled differently by the immune system with the development of parallel but separate mechanisms for dealing with these foreign challenges. Intracellular antigens produce processed peptides (usually eight or nine amino acids in length), which are generally presented to CD8+ cells (T-suppressor/cytotoxic lymphocytes) by MHC class I molecules. Extracellular antigen is processed for presentation as 15–25 amino acid length peptides to CD4+ cells (T-helper cells) by the MHC class II molecules found on specialized (so-called professional) antigen-presenting cells, such as dendritic cells, macrophages or B lymphocytes. The mechanisms of MHC-restricted antigen processing are shown in Figure 1.

Figure 2 shows the complex activation process of the T cell receptor.

Tumour cells have developed several mechanisms to inhibit immunological attack for survival advantage in hostile locations.

The failure of a tumour to induce a specific rejection response may partly be a result of the poor expression of foreign TAAs, such as CEA. Knowledge of the intricate mechanisms involved in foreign antigen presentation has permitted the development of potential genetic targets to overcome heterogeneity of surface antigen expression. The discovery of co-stimulatory signals has created a model of lymphocyte and specifically TcR activation (Bretscher and Cohn, 1970), whereby processed antigen may be linked either to the MHC complex or to a co-stimulatory ligand in its presentation to the T cell.

Co-stimulatory signals implicated in the activation of the TcR include tyrosine kinases, interleukins, the B7 family (Chen et al, 1993), the ICAM group, lymphocyte function-associated antigens, vascular cell adhesion molecules (VCAM-1) and heat stable antigens (HSA).

Studies of immunological tolerance have also assisted in the understanding of mechanisms of tumour escape from immune

© Cancer Research Campaign 1998

surveillance and lysis. The exposure of both the T- and the B-cell lineage to TAAs may result in clonal selection, with proliferation of immunocompetent effector cells, clonal anergy with down-regulation of immunogenic capacity or effector maturation arrest (Nossal and Pike, 1980; Goodnow et al, 1991). The concept of a population of down-regulated T- and B-cell repertoires with a threshold affinity for silencing was advanced by Nossal (1983) as one of 'immune ignorance'. T-cell tolerance of this type, which is part of normal thymocyte maturation of medullary CD4+ and CD8+ cells, permits the acquisition of TcR molecules with a high affinity for self-MHC and self-epitope recognition (Miller and Moralan, 1992; Shortman, 1992). How important this model is, however, outside in vitro systems is not known. Immune ignorance as opposed to T-cell deletion or anergy appears to be a more complex phenomenon and is secondary to a differential inability of lymphocytes in the periphery to recognize antigenic motifs in restricted tissue sites.

B-cell tolerance is clearly physiologically important too, as it prevents the development of a range of naturally produced auto-antibodies against cross-reactive self-epitope. This type of auto-antibody phenomenon, although common, is fortunately transient. The decision between clonal anergy and clonal ignorance is probably dependent upon the affinity of the B-cell receptor for the antigen concerned as well as the antigenic molar concentration. Anergy is favoured in states of very high antigen concentration, strong antigen cross-linking and high antibody affinity.

Widespread extracellular antigen expression (of CEA, for example) may therefore have already induced substantial T- and B-cell tolerance of the types mentioned. As a result, any anti-tumour vaccine based upon a native protein needs to either couple the epitope for recognition with another highly immunogenic carrier molecule (a so-called adjuvant) or use secondary strategies to enhance its immunogenicity.

Intratumoral variation may also provide an avenue for tumour escape from immunological attack. Previously, the main markers for tumour heterogeneity were morphological, biochemical and karyotypic, but increasingly there is recognized to be both molecular biological and immunohistochemical variability within tumour cell subpopulations that may affect immunotherapeutic and chemotherapeutic response. The multistep nature of colorectal carcinogenesis postulates potential mechanisms for intratumoral heterogeneity. At its simplest level, differences in tumour differentiation and tumour DNA ploidy may be reflected in differences in outcome. Tumour aneuploidy has been shown to correlate with overall prognosis in ovarian, renal cell, thyroid, adrenal and breast cancer (Rodenburg et al, 1987; Hamming, 1988; Oosterwijk et al, 1988; Haak et al, 1993; Hedley et al, 1993).

Finally, tumour heterogeneity may be a reflection of the underlying host immune defence systems. Potential mechanisms for immunological escape by tumour cells include changes in the structure of crucial molecules, such as MHC activation ligands, regulators of complement activation, lytic enzyme neutralizers and adhesion molecule receptors.

Disturbances in B-cell-mediated responsiveness may occur through other mechanisms, such as insufficient neoantigen presentation, relative immunological isolation (in areas such as the central nervous system or in ocular tumours), tumour-produced suppression by local inhibitors (prostaglandins and transforming growth factor beta) and drug-induced immunosuppression. In this sense, the immune system contributes to the phenotypic heterogeneity of the tumour.

**Figure 3** Representation of the carcinoembryonic antigen family. Ig-like domains are shown as ribbons. The black N domain is Ig v-like and the striped domains are Ig C-like. Ig C regions are repeated extracellularly into discrete domain regions A1–3 and B1–3. In the CEA molecule the membrane attachment is by a glycosyl phosphatidyl inositol anchor. Biliary glycoproteins (BGP) are membrane attached by a hydrophobic transmembrane region with a relatively long intracytoplasmic tail for signal transduction. Pregnancy-specific globulins (PSG) are actively secreted from the cell. GPi, glycosyl phosphatidyl inositol hook; Tm, ... transmembrane attachment

### CEA AS AN IMMUNOGENIC TARGET: STRUCTURE AND FUNCTION

Knowledge of the molecular structure of CEA defines recognition epitopes as immunological targets and assists in the design of monoclonal antibody therapy directed against cell-based TAA. This is particularly important in a molecule such as CEA, in which extensive carbohydrate moieties mask epitopes.

Molecular cloning at the cDNA level has permitted the identification of at least 29 CEA-related genes that are tightly clustered on a 1.2-Mb region located on the long arm of chromosome 19 (Tynan et al, 1992).

Comparisons of related family members have shown high sequence conservation (80–95% of N domain exons within subgroups and 65–70% homology between subgroups). The CEA gene along with that for non-specific cross-reacting antigen (NCA) and the biliary glycoproteins (BGP) comprise the CEA subgroup and the pregnancy-specific globulins (PSG) the other group of related genes. The basic common domain structure of the CEA subgroup (Figure 3) incorporates a variable number of internal repeating subunits that have equivalent secondary and tertiary structure to the immunoglobulin $C_2$ domains. The N domain of the molecule is structurally homologous, with the immunoglobulin V-like domain rendering the CEA family within the immunoglobulin superfamily of molecules (Williams and Barclay, 1988).

CEA is attached to the cell membrane by a glycosyl phosphatidylinositol (GPi) hook that is structurally distinct from the transmembrane attachment of the PSG group member proteins (Hefta et al, 1988; Thompson and Zimmerman, 1988; Ferguson, 1991; Thompson et al, 1991).

The homology of CEA with basic immunoglobulin molecules gives a clue as to the functional role of CEA-related antigens. Those containing $C_2$ domains are involved in cell adhesion. CEA-transfected cells have been shown to have adhesive properties through both homophilic and heterophilic interactions. Selective binding between carbohydrate moieties on CEA and lectin molecules on bacterial fimbriae may permit CEA to regulate gut–bacterial binding and control lymphocyte homing during gut

© Cancer Research Campaign 1998 

inflammation (Leusch et al, 1990). As a result, the regular luminal shedding of CEA may serve to control luminal bacterial load and translocation across the gut.

Many studies with regard to differential expression of CEA-related proteins during development have been produced in animal models that do not normally possess endogenous CEA-like species. The specificity of both polyclonal and monoclonal antibodies to CEA for immunohistochemistry has been enhanced by the use of cDNAs in expression vector systems producing a series of stable transfectant eukaryotic clones expressing most of the major CEA-related protein products (Arakawa et al, 1990; Berling et al, 1990; Hefta et al, 1990).

Over 50% of the CEA molecule is glycosylated with at least 28 separate putative sites of carbohydrate attachment. The position and density of these sites affect the exposure of potential antigenic recognition surfaces and, because of the heavy glycosylation of the molecule, crystallization for diffraction studies and structure interpretation have been difficult (Bates et al, 1992). Knowledge of these binding sites will permit the engineering of antibody molecules and other immune targeting agents based on CEA epitope structure that have slower off rates and that facilitate tumour retention by the antibody molecule (Boehm et al, 1996).

Limitations should be placed on extrapolation of data regarding the inherent immunogenicity of CEA within such models. For this work, it has been necessary to introduce CEA gene-regulatory plus indicator segments into transgenic mice as well as to produce gene inactivation by homologous recombination in induced colonic tumours that subsequently express human CEA (Eades-Perner and Zimmerman, 1995).

Recently, the novel anti-CEA monoclonal antibody PR1A3 has been successfully used in radioimmunoscintigraphy for the detection of CT-negative and CEA-negative recurrent colorectal cancer as well as to investigate the cell-based epitope domain of CEA (Granowska et al, 1989, 1993; Durbin et al, 1994). This antibody was originally produced in mice against components of normal human colonic epithelium and has been demonstrated to be highly sensitive by immunohistochemistry for nearly all human colorectal carcinomas, regardless of differentiation. It is highly specific with only minor cross-reactivity in normal respiratory epithelium (Richman and Bodmer, 1987). The significance of this antibody is its inability to bind circulating and purified CEA or CEA released from tumours and sequestered in lymph nodes. This epitope is not expressed by bacteria transfected with CEA fusion genes, implying the importance of post-translational modification and/or conformational changes in CEA once shed. The use of CEA-BGP chimaeric peptides has localized the PR1A3 epitope to the C terminus of the protein and binding is entirely reliant upon the presence of a small spacing peptide between the GPi anchor mechanism and the BGP recombinant construct (LM Stewart and D Snary, personal communication). This spacing peptide is believed to lift the B3 domain away from the cell membrane and permit antibody binding to the epitope. The exact mechanism of abrogated binding to circulating antigen is unknown, but may involve partial domain loss, the formation of steric hindrance by dimers of CEA or disruption of the epitope on release from the cell.

Immunotherapeutic strategies making use of murine (and humanized) PR1A3 will have the advantage of providing surrogate responses to cell-based TAA, avoiding circulating complex formation. At present, a phase I/II trial using murine PR1A3 in chemo-relapsed colorectal cancer has commenced at our institution.

### HUMORAL AND CELL-MEDIATED RESPONSES TO CEA

There is conflicting evidence that CEA functions as a natural immunogen in patients with advanced epithelial malignancy. It has traditionally been supposed that CEA is likely to have induced a state of tolerance and that this is compounded by the relative anergy evident in patients with advanced disease (Monson et al, 1986).

Some studies have consistently demonstrated the presence of circulating specific anti-CEA antibodies by indirect haemagglutination (Gold, 1967), radioimmunoassay (Gold et al, 1972; MacSween, 1975) and affinity chromatography (Pressman et al, 1980). Moreover, specific immune complexes directed against CEA, when present, have been shown to inversely correlate with overall survival and disease stage (Kapsopulou-Dominos and Anderer, 1979; Staab et al, 1980; Mavligit et al, 1983; Ura et al, 1985; Konstadoulakis et al, 1994). Other groups (Collatz et al, 1971; LoGerfo et al, 1972; Sorokin et al, 1973) have been unable, however, to demonstrate CEA-specific antibodies in the sera of patients with different gastrointestinal neoplasms. Very early studies have shown CEA as non-stimulatory for autologous lymphocytes in in vitro blastogenesis assays (Lejtenyi et al, 1971; Hollinshead et al, 1972; Mavligit et al, 1973*a*), although these reports are difficult to interpret as there are a mixture of potentially anergy-inducing factors inherent in these experiments. Differences in tumour antigen extraction technique, inactivation of CEA during the extraction process and the potential need for presensitized lymphocytes in stimulation assays may all affect the outcome of results (Mavligit et al, 1973*b*).

Recently, molecular cloning techniques have identified a range of tumour-specific peptides (largely in malignant melanomas) that are recognized by autologous MHC-restricted human T cells. It is uncertain whether these peptides are actually normally processed in vivo or whether cytotoxic-specific T-cell repertoires to these agents naturally exist (Slingluff et al, 1993; Wolfel et al, 1994).

Given the recent evidence of natural CTL reactivity against the normal tyrosinase enzyme system in melanoma patients, it is likely that CEA may be sufficiently immunogenic either alone or antigenically enhanced to function as a cancer vaccine (Anichini et al, 1993). Similar MHC-restricted CTLs have been demonstrated in ovarian and renal carcinoma, sarcoma, squamous cell carcinoma of the head, neck and lung and glioblastoma (Miyatake et al, 1986; Slovin et al, 1986; Ioannides et al, 1991, 1993; Finke et al, 1992). It remains unclear why natural tolerance to these peptides is not fully established or why T cells become reactive to self peptides on melanoma, for example, but the same peptides are not recognized by the lymphocytes of patients bearing other cancer histologies.

The potential options for using CEA as a direct immunizing antigen include the use of recombinant vaccinia virus–CEA constructs, polynucleotide CEA vaccination and anti-idiotypic antibodies. Secondary strategies rely on recombinant CEA and CEA-derived peptide booster therapy to maintain specific anti-CEA response.

### RECOMBINANT VACCINIA CEA (rV-CEA)

The strategy here is that a relatively weak immunogen is presented with a highly immunogenic viral protein and that the resultant

 © Cancer Research Campaign 1998

immune reaction is directed in part against the inserted gene product (Kaufman et al, 1991).

The insertion of stable eukaryotic genes into vaccinia vectors is only a recent development (Edwards and Rutter, 1988). The vaccinia virus is capable of co-presentation of antigen, and constructed vaccinia viruses have been shown to protect animals against infectious disease and tumour challenges (Bennick et al, 1984; Moss et al, 1984; Bernards et al, 1987; Lathe et al, 1987; Moss and Flexner, 1987; Estin et al, 1988). It has been shown that vaccinia virus vectors are stable and that inserted gene products from human colon cancer cell libraries are expressed and normally post-translationally modified (Coupar et al, 1988). Preliminary work has shown the induction in mice of specific anti-CEA antibodies, with reduction in the growth pattern of syngeneic murine colon carcinoma deposits transduced with the human CEA gene.

RV-CEA also induces CEA-specific lymphoproliferative and CTL responses as well as delayed-type hypersensitivity reactions. (DTH) (Kantor et al, 1992*a*). The virus insert approach has also been successfully used in mouse and primate tumour models against the melanoma-associated antigen p97, which is weakly expressed on normal cells, and this has resulted in subsequent protection against tumour challenge with cells expressing the human *p97* gene product (Estin et al, 1988; Hu et al, 1988). Recombinant vaccinia (and other virus) products will not be perfect, however, as there are cross-reactive epitopes for CEA-like species, such as NCA, normally expressed on human (and primate) granulocytes. The hope for clinical use is that immune responses principally occur to the immunodominant epitope located on CEA and that immunotolerance may be greater to the more widely distributed NCA antigen (Nap et al, 1988).

The results of the use of this approach in a rhesus monkey model that displays primate MHC and in which NCA cross-reacting antigen is expressed on normal monkey granulocytes show that it induces proliferative DTH response to intradermal challenge with CEA, proliferative blastogenesis to CEA and also primate-directed antibody-induced lysis of CEA-bearing tumour cells using human effector lymphocytes. The treatment has been shown to be relatively free of side-effects (Kantor et al, 1992*b*). In humans, recombinant vaccinia viruses have been shown to be safe, stable and to have acceptable immunogenicity, even when the individual has been previously exposed to a vaccinia virus as occurs after routine smallpox vaccination (Karzon, 1985; Chelyapov et al, 1988).

A phase I clinical trial has been reported by Hamilton et al (1994) in 26 patients with gastrointestinal, lung and breast cancers using $10^7$ plaque forming units (p.f.u.) of rV-CEA at monthly intervals for 3 months. T-cell responses to the vaccinia virus were observed, but there was no response to soluble CEA in blastogenesis assays.

Canarypox (Avipox group) has also been engineered to express the human cDNA of CEA. This virus is restricted, however, in its replication hosts, although it is likely to result in enhanced immunoresponsiveness in those patients previously exposed to smallpox vaccination or when local reactivity to repeated rV-CEA proves to be unacceptable (Hodge et al, 1997).

Antigenic peptides reflecting potential class I epitopes of CEA have recently been selected by screening for matches to consensus motifs of HLA-A2 and A3 binding peptides as the most commonly expressed HLA alleles. The CEA peptides (so-called CAP peptides) identified have been incubated in a T-cell binding assay in which up-regulation of surface HLA-A2 on the T cells was quantified by flow cytometry using an anti HLA-A2 antibody label (Nijman et al, 1993).

Specific T-cell lysis has been generated against autologous EBV-transformed B cells presenting the CAP-1 peptide motif but not against autologous non HLA-A2 EBV-transformed B cells pulsed with the same peptide. Tumour cell lysis of lines transduced with CEA serve as targets for these effector cells, implying that autologous B cells present and process these antigens in an MHC-restricted fashion. Allogeneic SW403 HLA-A2-positive cell lines also expressing CEA function as equivalent targets and non HLA-A2 allogeneic carcinoma cell lines (SW 1417 and HT-29) that do not express substantial CEA are not lysed. This is the first study to demonstrate peptide based CEA-specific CTLs and evidence of MHC-restricted CEA epitope processing by B cells. (Conry et al, 1995*a*).

This type of therapy still requires substantial work. The importance of non-human CEA-like and human CEA-transduced systems in natural immunity is unclear. Epitopes that are immunologically relevant in tumour biology must be able to be stably and consistently coexpressed with dominant immunogenic viral peptides. Tachyphylaxis associated with such approaches still needs to be overcome, but it is evident that troublesome cross-reactivity does not appear to be a clinical problem.

### CEA POLYNUCLEOTIDE VACCINATION

This form of active specific immunotherapy may be provided by both DNA and RNA and has certain advantages over tumour cell vaccines. It avoids potentially replicating virus and the need for adjuvants and appears stable in terms of gene product expression and induction of CEA-specific T-cell repertoires. Intracellular synthesis of the TAA favours MHC class I display and large quantities of the vaccine can in theory be produced and standardized for clinical use (Wolff et al, 1992; Conry et al, 1994, 1995*b*, 1996*a*).

The use of this form of immunization avoids potential recombinational events that may produce replication-competent viruses or the inadvertent incorporation of viral genomes into the host chromosomal complement. Both of these events may have serious consequences from the standpoint of the activation of oncogene sequences. The direct delivery of naked DNA therapy will also reduce the likely event of insertional mutagenesis. Polynucleotide vaccination uses the full length of h CEA cDNA driven by a CMV promoter and induces anti-CEA humoral and CEA-lymphoproliferative responses in mice. It has not been shown, however, to result in murine protection against syngeneic challenge with CEA-transduced colorectal cancer cells, unless administered by the intramuscular route. Stable gene expression systems use murine intramuscular plasmid injection, and DNA-coated bead projectiles have also been developed (Wolff et al, 1990; Yang et al, 1990).

The level of immune responsiveness with DNA vaccination appears equivalent to that induced by rV-CEA, although there is greater dose and schedule dependency. The amounts of gene gun dosage required appear to be minute (Eisenbraun et al, 1993; Pertmer et al, 1997).

The system of direct intramuscular plasmid injection needs improvement as mouse myocytes expressing CEA tend to die after about 10 days. The mechanism whereby the mouse myocyte functions as a semiprofessional antigen presenting cell is at present unknown, however myocytes have been shown to up-regulate MHC expression after γ-interferon stimulation, and their immunostimulant capacity is enhanced by co-transfection with B7

© Cancer Research Campaign 1998

**Figure 4** The immune network in tumour biology. A range of anti-idiotypic antibodies are produced in response to the $AB_1$ molecule. $Ab_2\beta$ antibodies recognize the antigen binding site of $Ab_1$ and are 'internal images' of the epitope. $Ab_2\alpha$ antibodies recognize idiotopes that lie outside the antigen binding site of $Ab_1$. $Ab_2\gamma$ antibodies recognize a portion of the antigen binding site of $Ab_1$ but do not carry the internal image of the antigen. An idiotype cascade results in the production of $Ab_3$ antibodies that resemble $Ab_1$ in their binding site sequence and that secondarily recognize the antigen

**Table 1** Techniques to enhance monoclonal antibody tumour targeting

| |
|---|
| *Strategies to enhance tumour localization* |
| Vascular endothelial monoclonal antibodies |
| New labelling techniques |
| Different antibody isotypes |
| Genetically engineered antibodies |
| High-affinity antibodies |
| Unlabelled antibody pre-dosing |
| Antibody cocktails |
| Fractionation |
| *Strategies to increase radioimmunoconjugate clearance* |
| Antibody fragments |
| Pre-targeting approaches |
| Metabolizable chelates |
| Second-clearing antibodies |
| Plasmapheresis |
| *Other localizing strategies* |
| Regional administration |
| Biological-response modifiers to increase TAA expression |
| Increases in tumour vascularity |

plasmids, suggesting a role as formal antigen presenters (Goebels et al, 1992; Hohlfield and Engel, 1994; Conry et al, 1996*b*). It may be that an inflammatory response against the plasmid construct evokes a secondary recruitment of professional antigen-presenting and other effector cells.

Successful CEA polynucleotide vaccination strategies have now been conducted in non-human primates (Wang et al, 1993). An initial phase I dual-specific plasmid DNA (CEA and hepatitis B surface antigen) study in patients with metastatic colorectal cancer has recently been jointly approved by the Recombinant DNA Advisory Committee and the NIH in the USA (Conry et al, 1996*c*). The use of naked mRNA vaccination offers several advantages when injection of DNA potentially encoding tumour growth factors risks incorporation of delivered genetic material into the host genome. This approach has been used with CEA mRNA transcripts transfected into CEA-negative cell lines using cationic liposome vectors. In this instance, the immunization schedule to maintain CEA expression needs to be more intensive than that used for naked DNA. Most mice, however, develop anti-CEA antibody responses when challenged with CEA-expressing tumour cells. Work is progressing using single-stranded RNA vectors for eukaryotic transfection that undergo self-replication after transduction but that are non-infectious (i.e. they do not contain the gene regions encoding viral-packaging proteins). So far, representatives of the Togaviridae have been used, notably poliovirus, Semliki Forest virus and Sindbis virus (Ansardi et al, 1994; Conry et al, 1995*c*; Zhou et al, 1995).

The in vivo delivery of plasmid DNA encoding a relevant TAA, although immunologically effective in animal models, has several problems in humans. The principal difficulty is the reduced efficiency of non-human primate and human expression of plasmid DNA in muscle (Jiao et al, 1992). This may require the co-delivery of either a cytokine gene or co-stimulator cDNA for enhancement of the CEA polynucleotide.

### DESIGNER MONOCLONALS, ANTI-IDIOTYPIC ANTIBODIES AND COLORECTAL CANCER

Jerne's proposal of a cascading network of idiotype–anti-idiotype antibodies after antigenic immunization (Jerne, 1974) has been widely adopted as a model for the induction of V-domain interactions as part of a humoral response to available TAAs. This immune network is shown in Figure 4. The primary antibody ($Ab_1$) reacting with the main antigenic epitope contains components on its V domain that function as secondary 'epitopes' and that are recognized as antigenic by a second line of antibody molecules referred to as anti-idiotypic ($Ab_2$) antibodies. These $Ab_2$ molecules are serologically and stereochemically divisible into several subgroups: $Ab_2\alpha$ molecules, which identify idiotopes outside the antigen binding site; $Ab_2\beta$ molecules, which function as internal images of the original antigen; and $Ab_2\gamma$ molecules, which are capable of partial antigen/$Ab_1$ blockade but which are not antigenic internal images. Anti-idiotypic antibodies potentially induced by TAA exposure may serve as natural agents for use in passive immunotherapy because of their ability to act as surrogate antigen vaccines, being capable of stimulating anti-anti-idiotypic antibodies (designated as $Ab_3$), which functionally mimic the steric structure of the $Ab_1$ molecule and which directly attack the primary antigen.

Such anti-idiotypic cascades are involved in immune regulation in several ways. $Ab_2$ antibodies are capable of neutralizing circulating $Ab_1$ as well as binding to surface immunoglobulin receptors on activated B cells, thus interfering with TcR function and T-/B-cell interaction.

Monoclonal antibodies are used in patients with colorectal cancer for radioimmunolocalization of recurrent or metastatic disease, in radioimmunoguided surgery and as specific immunotherapy, inducing anti-idiotypic cascades and CTL reactivity when directed against well-characterized epitopes of CEA. The latter approach is undergoing a revolution with the use of humanized and bifunctional antibodies, F(ab′) and single Fv fragments as well as with human anti-idiotypic antibodies. In addition, therapies may be conjugated with toxins or radiopharmaceuticals.

There are many barriers to monoclonal antibody usage in solid malignancy. Heterogeneity of TAA and MHC expression may limit antibody binding. Physical factors most notably related to distorted tumour vascular architecture and increased intratumoral interstitial pressure limit the diffusion and biodistribution of macromolecules to the periphery of tumour deposits.

As tumour deposits enlarge, available surface area for transvascular molecular exchange diminishes in the majority of tumour types (Jain et al, 1988). Further, as the interstitial pressure

 © Cancer Research Campaign 1998

particularly in the centre of most tumours exceeds that of normal tissue, convection of macromolecules through the interstitial space, which is primarily dependent upon pressure gradients between the vascular and extravascular spaces, will work against the movement of antibodies towards the tumour matrix (Jain, 1987).

Approaches to overcome these difficulties are shown in Table 1. Lower-molecular-weight fragments of the primary antibody or the regional administration of antibody may improve local intratumoral concentration, but are often associated with accelerated elimination. The pharmacokinetics of these agents as well as that of bifunctional antibodies with hypervariable murine anti-idiotypic domains linked to TcR cell surface recognition molecules, remains to be elucidated.

One of the greatest difficulties with the use of murine monoclonal antibodies is the development of a human anti-murine antibody (HAMA) response to the Fc portion of the primary mouse antibody administered. The extent of this response particularly to repeated murine exposure will limit the therapeutic effect of monoclonal treatment, shorten antibody half-life, enhance clearance of antibody and potentially induce a serum sickness reaction in treated patients. The nature of the HAMA response is polyclonal with anti-isotypic and anti-idiotypic reactivity and may even affect the administration of human and humanized antibodies. This type of heterophilic antibody response will also interfere with assays that routinely use murine monoclonals, most notably standard CEA assay (Morton et al, 1988).

The finding of significant HAMA responses has resulted in the production of a range of designer antibodies, such as chimaeric antibodies, $V_H$ domain molecules, antigen-binding peptides and recombinant antibody fusion proteins (Mayforth and Quintans, 1990; Fell et al, 1991; Winter and Milstein, 1991).

Genetically engineered antibodies that lack Fc reactivity could be used when Fc function is not desired, such as in radioimmunolocalization to diminish background (Bird et al, 1988). Single-chain antigen-binding fragments (sFv) and recombinant sFv peptides, which consist of $V_L$ and $V_H$ domains joined by peptide linkers and expressed in large quantity by *Escherichia coli*, are being developed for imaging purposes, although problems exist both with reduced affinity compared with the parent molecule and steric hindrance of the linker peptides. Many of these newer peptides are also relatively unstable. Despite chimaerization, anti-idiotypic antibodies that recognize the murine V region are potentially still a problem (Bruggemann et al, 1989). Although humanization of antibodies reduces their immunogenicity, the antigen–antibody binding affinity of the parent antibody may not be reproduced.

Although idiotypic–anti-idiotypic cascades can be demonstrated in patients with tumours after xenogeneic monoclonal antibody therapy, their exact significance is not known. The further advantage of such immune therapy, however, in solid tumours is their relative ease of production for general use, without the need for custom-made therapies using autologous tumour cells or autologous-stimulated TILs. The recent development of genetic recombinant libraries expressing specific epitope domains that are entirely human will enhance the ability to expand the repertoire of immunotherapies against a variety of unique TAAs, with large-scale production of antibody for use in conjunction with either conventional chemotherapy or progenitor cell support (Bona, 1989).

One of the main advantages of using anti-idiotypic antibodies in tumour therapy is in states in which the primary antigen is either weakly expressed or is frankly non-immunogenic. They may, in theory, break immune tumour tolerance to weak determinants and be useful against antigens that are difficult to characterize or synthesize. The use of human anti-idiotype therapy rather than either monoclonal or polyclonal xenogeneic anti-idiotype therapy avoids troublesome interspecies reactivity and the induction of inappropriate and non-specific human T-cell repertoires. Human therapies are likely to mediate more efficient complement-dependent cell lysis and ADCC (Chattopadhayay et al, 1992; Koido et al, 1995). For anti-idiotypes, the problems of oversecretion of complexing antibody, HAMA responsiveness, the induction of down-regulating idiotypic cascades and the difficulty of matching bizarre tumour cell idiotopes that are not shared between tumour types still remain. Human anti-idiotypic monoclonal therapy has resulted in survival benefit in patients with advanced malignant melanoma, and advanced colorectal carcinoma has demonstrated improved outcome when compared with historical controls (Robins et al, 1991*a*; Mittelmam et al, 1992).

Further, CTL activity of both peripheral blood and mesenteric node lymphocytes against autologous tumour has been demonstrated in a small number of patients with rectal cancer after immunization with human anti-idiotypic antibody when lymphocytes did not initially respond in vitro to autologous biopsy material (Austin et al, 1991; Durrant et al 1994*a* and *b*; Robins et al, 1991*b*). Similar findings producing anti-CEA antibodies have been shown in cynomolgus monkeys using the murine anti-idiotypic antibody 3H1, which mimics an epitope on CEA normally absent on adult colonic epithelium (Bhattacharya-Chatterjee et al, 1990; Chakraborty et al, 1995).

There is as yet comparatively poor prediction of the relative immunogenicity of anti-idiotypes necessary for T-cell responsiveness in tumour systems (Raychaudhuri et al, 1990; Tsang et al, 1995). Recently, cytokines have been used in combination with monoclonal antibodies to enhance MHC (Rosa and Fellous, 1988) and CEA (Kantor et al, 1989) expression as well as to improve residual tumour radioimmunodetection (Nieroda et al, 1995). Interferon-γ has been shown to increase CO 17-1A-directed ADCC by human effector cells against colorectal cancer cell lines (most notably SW 116) (Steplewski et al, 1986); however, phase II studies in patients with advanced colorectal carcinoma combining the monoclonal antibody 17-1A with interferon-γ, although safe for clinical use, have shown inconsistent anti-idiotypic responsiveness and poor clinical responsiveness (Blottiere et al, 1990). Granulocyte–macrophage colony-stimulating factor (GM-CSF) (Sieff et al, 1985), which stimulates differentiation and maturation of the monocyte–macrophage lineage, enhances in vitro ADCC function, stimulates delayed-type hypersensitivity and encourages professional antigen presentation, has also been used in combination with monoclonal antibody therapy (Morrissey et al, 1987). Recent reports assessing its use in combination with 17-1A in advanced colorectal cancer have shown clinical remissions, although the therapy is marred in some patients by the presence of immediate-type allergic responses to the murine monoclonal after repeated exposure. This has necessitated reduction of the monoclonal antibody dose (Raganhammar et al, 1993, 1995). Further, the ultimate development in most patients of neutralizing anti-GM-CSF antibodies after combination therapy may result in significant failure of the normal peripheral lymphocyte expansion seen during colony-stimulating factor (CSF) treatment.

This may have a signicant bearing on the type and level of sustainable immune response during monoclonal antibody treatment. This is particularly evident in non-immunosuppressed patients capable of mounting an auto-immune reaction against endogenous colony stimulating proteins. This may render conventional CSF therapy in such patients relatively ineffective (Wadhwa et al, 1996). It is clear that unconjugated anti-idiotypic therapy induces specific humoral and cell-mediated responses against syngeneic and histocompatible colorectal cancer cell lines. At present, the dosage scheduling and the need for combination therapies in patients with advanced disease has yet to be determined. Clinical responses to date are sporadic.

### FUTURE STRATEGIES OF BIOLOGICAL THERAPY AND COLORECTAL CANCER

The prospects for gene therapy in colorectal cancer include the correction of abnormal oncogenes implicated in the development of colorectal tumours, the augmentation or replacement of tumour-suppressor genes, such as *p53*, and strategies to interfere with tumour-related growth factor and growth factor receptor genes.

Ancilliary approaches will include genetically directed immunopotentiation of effector lymphocytes either to improve TIL capacity or to encourage TIL homing to tumours. The possible exploitation of techniques directed at newly discovered angiogenic factors controlling tumour neovasculature represents an exciting potential therapy (Baillie et al, 1995).

Tumour cells themselves may also be transduced with cytokine and cytokine receptor genes to render them suitably immunogenic.

The gastrointestinal tract represents a unique portal for potential gene therapy, although new techniques of delivery, such as the use of liposomal carriers, biodegradable microspheres and attenuated *Salmonella* spp. carriers, are required for consistent gene expression in such a hostile environment.

The Fearon–Vogelstein model of colorectal tumorigenesis (Fearon and Vogelstein, 1990) represents a challenge to modify the natural history of colonic tumours and premalignant disease through genetic intervention, as it is recognized that many mammalian cells have the cellular machinery required for successful integration of foreign genetic material into the parent genome (Capecchi, 1989). Many of these approaches in colorectal cancer are still theoretical, and much work needs to be done before they can become clinically valuable. The approach to increase the immunogenicity rather than the antigenicity of the tumour cell itself and to abrogate a tumour-induced immunosuppressive microenvironment remains a significant challenge for the future.

### REFERENCES

Adams DO, Hall T, Steplewski Z and Koprowski H (1984) Tumours undergoing rejection induced by monoclonal antibodies of the $IgG_{2A}$ isotype containing increased numbers of macrophages activated for a distinctive form of antibody dependent cytolysis. *Proc Natl Acad Sci USA* **81**: 3506–3510

Anichini A, Maccalli C, Mortarini R, Salvi S, Mazzochi A, Squareina P, Herlyn M and Parmiani G (1993) Melanoma cells and normal melanocytes share antigens recognized by HLA-A2 restricted cytotoxic T cell clones from melanoma patients. *J Exp Med* **177**: 989–998

Ansardi DC, Moldoveanu Z, Porter DC, Walker DE, Conry RM, LoBuglio AF, McPherson S and Marrow CD (1994) Characterization of poliovirus replicons encoding carcinoembryonic antigen. *Cancer Res* **54**: 6359–6364

Arakawa F, Kuroki M, Misumi Y, Oikawa S, Nakazato H and Matzuoka Y (1990) Characterization of a cDNA clone encoding a new species of the non-specific cross-reacting antigen (NCA) a member of the CEA gene family. *Biochem Biophys Res Commun* **166**: 1063–1071

August DA, Ottrow RT and Sugarbaker PH (1984) Clinical perspectives on human colorectal cancer metastases. *Cancer Metastasis Rev* **3**: 303–324

Austin EB, Robins RA and Durrant LG (1991) Induction of delayed hypersensitivity to human tumour cells with a human monoclonal anti-idiotypic antibody. *J Natl Cancer Inst* **83**: 1245–1284

Baillie CT, Winslet MC and Bradley NJ (1995) Tumour vasculature – a potential therapeutic target. *Br J Cancer* **72**: 257–267

Bates PA, Luo J and Sternberg MJE (1992) A predicted three-dimensional structure for the carcinoembryonic antigen (CEA). *FEBS* **301**: 207–214

Bennick JR, Yewdell JW, Smith GL, Moller C and Moss B (1984) Recombinant vaccinia virus primes and stimulates influenza haemagglutinin specific cytotoxic T cells. *Nature* **311**: 578–579

Berling B, Kolbinger F, Grunert F, Thompson JA, Brombacher F, Buchegger F, von Kleist S and Zimmerman W (1990) Cloning of a carcinoembryonic antigen family member expressed in leukocytes of chronic myeloid leukemia patients and bone marrow. *Cancer Res* **50**: 6534–6539

Bernards R, Destree A, McKenzie S, Gordon E, Weiberg RA and Panicali D (1987) Effective tumour therapy directed against an oncogene-encoded product using a vaccinia virus vector. *Proc Natl Acad Sci USA* **84**: 6854–6858

Bhattacharya-Chatterjee M, Mukerjee S, Biddle W, Foon KA and Kohler H (1990) Murine monoclonal anti-idiotype antibody as a potential network antigen for human carcinoembryonic antigen. *J Immunol* **145**: 2785–2765

Bird RE, Hardman KD, Jacobson JW, Johnson S, Kaufman BM, Lee SM, Lee T, Pope SH, Riordan GS and Whitlow M (1988) Single-chain antigen-binding proteins. *Science* **242**: 423–426

Blottiere HM, Douillard J-Y, Koprowski H and Steplewski Z (1990) Humoral and cellular responses of colorectal cancer patients treated with monoclonal antibodies and interferon-γ. *Cancer Immunol Immunother* **32**: 29–37

Boehm MK, Mayans MO, Thornton JD, Begent RHJ, Keep PA and Perkins SJ (1996) Extended glycoprotein structure of the seven domains in human carcinoembryonic antigen by X-ray and neutron solution scattering and an automated curve fitting procedure: implications for cellular adhesion. *J Mol Biol* **259**: 718–735

Bona CA (1989) Idiotypic network theory and its implications in anti-tumour immunity. *Immun Cancer* **2**: 215–221

Bretscher PA and Cohn M (1970) A theory of self discrimination. *Science* **169**: 1042–1049

Bruggemann M, Winter G, Waldmann H and Neuberger MS (1989) The immunogenicity of chimaeric antibodies. *J Exp Med* **170**: 2153–2157

Capecchi MR (1989) Altering the genome by homologous recombination. *Science* **244**: 1288–1292

Chakaraborty M, Foon KA, Kohler H and Bhattacharya-Chatterjee M (1995) Preclinical evaluation in non-human primates of an anti-idiotypic antibody that mimics the carcinoembryonic antigen. *J Immunother* **18**: 95–103

Chattopadhayay P, Starkey J, Morrow WJW and Raychaudhuri S (1992) Murine monoclonal anti-idiotype antibody breaks unresponsiveness and induces a specific antibody response to human melanoma-associated proteoglycan antigen in cynomolgus monkeys. *Proc Natl Acad Sci USA* **89**: 2684–2688

Chelyapov NV, Antonova TP, Yanova NN and Chernos VI (1988) Antigenic properties of vaccinia virus and of the virus recombinant strains expressing heterologous genes. *Acta Virol* **32**: 409–416

Chen L, Linsley PS and Hellstrom KE (1993) Constimulation of T cells for tumour immunity. *Immunol Today* **14**: 483–486

Collatz E, von Kleist S and Burtin P (1971) Further investigations of circulating antibodies in colon cancer patients on the autoantigenicity of the carcinoembryonic antigen. *Int J Cancer* **8**: 298–303

Conry RM, LoBuglio AF, Kantor J, Schlom J, Loechel F, Moore SE, Sumerel LA, Barlow DL, Abrams S and Curiel DT (1994) Immune response to a carcinoembryonic antigen polynucleotide vaccine. *Cancer Res* **54**: 1164–1168

Conry RM, Saleh MN, Schlom J, Loechel F, Abrams S and Curiel DT (1995*a*) Breaking tolerance to carcinoembryonic antigen with a recombinant vaccinia virus vaccine in man. *Proc Am Assoc Cancer Res* 492(A)

Conry RM, LoBuglio AF, Loechel F, Moore SE, Sumerel LA, Barlow DL, Pike J and Curiel DT (1995*b*) A carcinoembryonic antigen polynucleotide vaccine for human cinical use. *Cancer Gene Ther* **2**: 33–83

Conry RM, LoBuglio AF, Wright M, Sumerel M, Pike MJ, Johanning F, Benjamin R, Lu D and Curiel DT (1995*c*) Characterization of a messenger RNA polynucleotide vaccine vector. *Cancer Res* **55**: 1397–1400

Conry RM, LoBuglio AF and Curiel DT (1996*a*) Polynucleotide mediated immunization therapy of cancer. *Semin Oncol* **23**: 135–147

Conry RM, Widera G, LoBuglio AF, Fuller JT, Moore SE, Barlow DL, Turner J, Yang N-S and Curiel DT (1996*b*) Selected strategies to augment polynucleotide vaccination. *Gene Ther* **3**: 67–74

Conry RM, LoBuglio AF and Curiel DT (1996*c*) Phase Ia trial of a polynucleotide

© Cancer Research Campaign 1998

anti-tumor immunization to human carcinoembryonic antigen in patients with metastatic colorectal cancer. Comprehensive Cancer Center University of Alabama Clinical Protocol. *Human Gene Ther* **7**: 755–772

Coupar BEH, Andrew ME and Boyle DB (1988) A general method for the construction of recombinant vaccinia viruses expressing multiple foreign genes. *Gene* **68**: 1–10

Durbin H, Young S, Stewart LM, Wrba F, Rowan AJ, Snary D and Bodmer WF (1994) An epitope on carcinoembryonic antigen defined by the clinically relevant antibody PR1A3. *Proc Natl Acad Sci USA* **91**: 4313–4317

Durrant LG, Doran M, Austin EB and Robins RA (1994*a*) Induction of cellular immune responses by a murine monoclonal anti-idiotypic antibody recognizing the 791 Tgp 72 antigen expressed on colorectal, gastric and ovarian human tumours. *Int J Cancer* **60**: 1–5

Durrant LG, Buckley TJD, Denton GWL, Hardcastle JD, Sewell HF and Robins RA (1994*b*) Enhanced cell mediated tumour cell killing in patients immunized with human monoclonal anti-idiotypic antibody 105 AD7. *Cancer Res* **54**: 4837–4840

Eades-Perner A-M and Zimmerman W (1995) Carcinoembryonic antigen transgenic mice: a model for tumour immunotherapy. *Tumour Biol* **16**: 56–61

Edwards RH and Rutter WJ (1988) Use of vaccinia virus vectors to study protein processing in human disease. *J Clin Invest* **82**: 44–47

Eichmann K and Rajewsky K (1975) Induction of T and B cell immunity by anti-idiotype therapy. *Eur J Immunol* **5**: 661–666

Eisenbruan MD, Fuller DH and Haynes JR (1993) Examination of parameters affecting the elicitation of humoral immune responses by particle bombardment mediated genetic immunization. *DNA Cell Biol* **12**: 791–797

Estin CD, Stevenson US, Plowman GD, Hu SL, Sridhar P, Hellstrom I, Brown JP and Hellstrom KE (1988) Recombinant vaccinia virus vaccine against the human melanoma antigen p97 for use in immunotherapy. *Proc Natl Acad Sci USA* **85**: 1052–1056

Fearon ER and Vogelstein B (1990) A genetic model for colorectal tumorigenesis. *Cell* **61**: 759–767

Fell HP, Gayle MA, Grosmaire L and Ledbetter JA (1991) Genetic construction and characterization of a fusion protein consisting of a chimaeric F(ab′) with specificity for carcinomas and human IL-2. *J Immunol* **146**: 2446–2452

Ferguson MAJ (1991) Glycosyl-phosphatidylinositol membrane anchors: the tale of a tail. *Biochem Soc Trans* **20**: 243–256

Finke JH, Rayman P and Edinger M (1992) Characterization of a human renal cell carcinoma specific cytotoxic CD8+ T-cell line. *J Immunother* **11**: 1–11

Goebels N, Michaelis D, Wekerle H and Hohfield R (1992) Human myoblasts as antigen presenting cells. *J Immunol* **149**: 661–667

Gold P (1967) Circulating antibodies against carcinoembryonic antigens of the human digestive system. *Cancer* **20**: 1663–1666

Gold JM, Freeman SO and Gold P (1972) Human anti-CEA antibodies detected by radioimmunoelectrophoresis. *Nature New Biol* **239**: 60–62

Goodnow CC, Brink R and Adams E (1991) Breakdown of self tolerance in anergic B lymphocytes. *Nature* **352**: 532–536

Granowska M, Jass J, Britton KE and Northover JMA (1989) A prospective study of the use of $^{111}$In-labelled monoclonal antibody against carcinoembryonic antigen in colorectal cancer and of some biologic factors affecting its uptake. *Int J Colorect Dis* **4**: 97–108

Granowska M, Britton KE, Mather SJ, Morris G, Ellison D, Soobramoney S, Talbot IC and Northover JM (1993) Radioscintigraphy with Tc$^{99m}$ labelled monoclonal antibody 1A3 in colorectal cancer. *Eur J Nucl Med* **20**: 690–698

Haak HR, Cornelisse CJ, Hermans J, Cobben L and Fleuren GJ (1993) Nuclear DNA content and morphologic characteristics in the prognosis of adrenocarcinomas. *Proc ASCO* 961(A)

Hamming JF, Shelfhout LJDM, Cornelisse CJ, Van de Velde CJH, Goslings BM, Hermans J and Fleuren GJ (1988) Prognostic value of nuclear DNA content in papillary and follicular thyroid cancer. *World J Surg* **12**: 503–508

Hardingham JE, Kotasek D, Farmer B, Butler RN, Mi JX and Dubrovic A (1993) Immunobead PCR: a technique for the detection of circulating tumour cells using immunomagnetic beads and the polymerase chain reaction. *Cancer Res* **53**: 3455–3458

Harlan DM, Abe R, Lee KP and Jane CH (1995) Short analytical review: potential roles of the B7 and CD 28 receptor families in autoimmunity and immune evasion. *Clin Immunol Immunopathol* **75**: 99–111

Hedley DW, Clark GM, Cornelisse CJ, Killander D, Kute T and Merkel D (1993) DNA cytometry consensus conference: consensus review of the clinical utility of DNA cytometry in carcinoma of the breast. *Breast Cancer Res Treat* **14**: 482–485

Hefta SA, Hefta LJF, Lee TD, Paxton RJ and Shively JE (1988) Carcinoembryonic antigen is anchored to membranes by covalent attachment to a glycosyl-phosphatidylinositol moiety: identification of the ethanolamine linkage site. *Proc Natl Acad Sci USA* **85**: 4648–4652

Hefta LFJ, Schrewe H, Thompson JA, Oikawa S, Nakazato H and Shively JE (1990) Expression of complementary DNA and genomic clones for carcinoembryonic antigen and nonspecific cross-reacting antigen in Chinese hamster ovary and mouse fibroblast cells and characterization of the membrane-expressed products. *Cancer Res* **50**: 2397–2403

Herlyn DM and Koprowski H (1981) Monoclonal anticolon cancer antibodies in complement dependent cytotoxicity. *Int J Cancer* **27**: 769–774

Hodge JW, McLaughlin JP, Kantor JA and Schlom J (1997) Diversified prime and boost protocols using recombinant vaccinia virus and recombinant nonreplicating avian pox virus to enhance T cell immunity and anti-tumour responses. *Vaccine* **15**: 759–768

Hohlfield R and Engel AG (1994) The immunobiology of muscle. *Immunol Today* **15**: 269–274

Hollinshead AC, McWright CG, Alford TC, Glew DH, Gold P and Herberman RB (1972) Separation of skin-reactive intestinal cancer antigen from the carcinoembryonic antigen of Gold. *Science* **177**: 887–889

Hu SL, Plowman GD, Sridhar P, Stevenson US, Brown JP and Estin CD (1988) Characterization of a recombinant vaccinia virus expressing human melanoma-associated antigen p97. *J Virol* **62**: 176–180

Ioannides CG, Freedman RS, Platsoucas CD, Rashed S and Kim YP (1991) Cytotoxic T cell clones isolated from ovarian tumour infiltrating lymphocytes recognize multiple antigenic epitopes on autologous tumour cells. *J Immunol* **146**: 1700–1707

Ioannides CG, Fisk B, Jerome KR, Irimura T, Wharton JT and Finn OJ (1993) Cytotoxic T cells from ovarian malignant tumours can recognize polymorphic epithelial mucin core peptides. *J Immunol* **151**: 5481–5491

Jain RK (1987) Transport of molecules in the tumour interstitium: a review. *Cancer Res* **47**: 3039–3051

Jain RK (1988) Determinants of tumour blood flow: a review. *Cancer Res* **48**: 2641–2658

Jerne NK (1974) Towards a network theory of the immune system. *Ann Immunol* **125(C)**: 373–389

Jiao S, Williams P, Berg RK, Hodgeman BA, Liu L, Repetto G and Wolff JA (1992) Direct gene transfer into non-human primate myofibers. *Hum Gene Ther* **3**: 21–33

Johnson PW, Burchill SA and Selby PJ (1995) The molecular detection of circulating tumour cells. *Br J Cancer* **72**: 268–276

Kantor J, Tran R, Greiner JW, Pestka S, Fisher PB, Shively JE and Schlom J (1989) Modulation of carcinoembryonic antigen messenger RNA levels in human colon carcinoma cells by recombinant human γ-interferon. *Cancer Res* **49**: 2651–2655

Kantor J, Irvine K, Abrams S, Kaufman H, DiPietro J and Schlom J (1992*a*) Antitumour activity and immune responses induced by a recombinant carcinoembryonic antigen vaccinia virus vaccine. *J Natl Cancer Inst* **84**: 1084–1091

Kantor J, Irvine K, Abrams S, Snoy P, Olsen R, Greiner J, Kaufman H, Eggensperger D and Schlom J (1992*b*) Immunogenicity and safety of a recombinant vaccinia virus vaccine expressing the carcinoembryonic antigen gene in a nonhuman primate. *Cancer Res* **52**: 6917–6925

Kantor J, Abrams S, Irvine K, Snoy P, Kaufman H and Schlom J (1993) Specific immunotherapy using a recombinant vaccinia virus expressing human carcinoembryonic antigen. *Ann NY Acad Sci* **690**: 370–373

Kapsopoulou-Dominos K and Anderer FA (1979) An approach to the routine estimation of circulating carcinoembryonic antigen immune complexes in patients with carcinomata of the gastrointestinal tract. *Clin Exp Immunol* **37**: 25–32

Karzon DT (1985) Considerations of safety, efficacy and potential applications of vaccinia vectors for immunoprophylaxis: an alternative approach for control of human disease for which vaccines are available. In *Vaccinia Viruses as Vectors for Vaccine Antigens*, Quinnan GV. (ed.), pp. 231–236. Elsevier: New York

Kaufman H, Schlom J and Kantor J (1991) A recombinant vaccinia virus expressing human carcinoembryonic antigen (CEA). *Int J Cancer* **48**: 900–970

King's Fund Forum (1990) Cancer of the colon and rectum. *Br J Surg* **77**: 1063–1065

Koido T, Scheck S and Herlyn D (1995) Induction of immunity to colon carcinoma antigen CO17-1A by monoclonal anti-idiotype ($Ab_2$): effects of $Ab_2$ fragmentation, carrier and adjuvant. *Tumor Targeting* **1**: 115–124

Konstadoulakis MM, Syrigos KN, Albanopoulos C, Mayers G and Golematis B (1994) The presence of anti-carcinoembryonic antigen (CEA) antibodies in the sera of patients with gastrointestinal malignancies. *J Clin Immunol* **14**: 310–313

Kradin RL, Lazarus DS and Dubinett SM (1989) Tumour-infiltrating lymphocytes and interleukin-2 in treatment of advanced cancer. *Lancet* **1**: 577–578

Lathe R, Kieny MP, Gerlinger P, Clertant P, Guizani I, Cuzin F and Chambon P

© Cancer Research Campaign 1998 

(1987) Tumour prevention and rejection with recombinant vaccinia. *Nature* **326**: 878–880

Lejtenyi MC, Freedman SO and Gold P (1971) Response of lymphocytes from patients with gastrointestinal carcinoma to the carcinoembryonic antigen of the human digestive system. *Cancer* **28**: 115–120

Leusch HG, Hefta SA, Drzeniek Z, Hummel K, Markos-Pusztai Z and Wagener C (1990) *Escherichia coli* of human origin binds to carcinoembryonic antigen (CEA) and non-specific cross-reacting antigen (NCA). *FEBS Lett* **261**: 405–409

Lindeman F, Schlimok G, Dirschel P, Witte J and Riethmuller G (1992) Prognostic significance of micrometastatic tumour cells in bone marrow of colorectal cancer patients. *Lancet* **340**: 685–689

LoGerfo P, Herter FP and Bennett FJ (1972) Absence of circulating antibodies to CEA in patients with gastrointestinal malignancies. *Int J Cancer* **9**: 341–344

MacSween JM (1975) The antigenicity of carcinoembryonic antigen in man. *Int J Cancer* **15**: 246–252

Mavligit GM and Stuckey S (1983) Colorectal carcinoma: evidence for circulating CEA–antiCEA complexes. *Cancer* **52**: 146–149

Mavligit GM, Gutterman JU, McBride CM and Hersh EM (1973*a*) Cell-mediated immunity to human solid tumours: in vitro detection by lymphocyte blastogenic responses to cell-associated and solubilized tumour antigens. *Natl Cancer Inst Monogr* **37**: 167–176

Mavligit GM, Ambus U, Gutterman JU and Hersh EM (1973*b*) Antigen solubilized from human solid tumours: lymphocyte stimulation and cutaneous delayed hypersensitivity. *Nature New Biol* **243**: 188–190

Mayforth R and Quintans J (1990) Designer and catalytic antibodies. *N Engl J Med* **323**: 173–178

Miller JFAP and Morahan G (1992) Peripheral T cell tolerance. *Ann Rev Immunol* **10**: 51–70

Mittelman A, Chen ZY, Yang H, Wong GY and Ferrone S (1992) Human high molecular weight melanoma-associated antigen (HMW-MAA) mimicry by mouse anti-idiotypic monoclonal antibody MK2-23: induction of humoral anti-HMW-MAA immunity and prolongation of survival of patients with stage IV melanoma. *Proc Natl Acad Sci USA* **89**: 466–407

Miyatake SM, Hanada H and Yamashita J (1986) Induction of human glioma specific cytotoxic T lymphocyte lines by autologous tumour stimulation and interleukin 2. *J Neurol Oncol* **4**: 55–64

Monson JRT, Ramsden C and Guillou PJ (1986) Decreased interleukin-2 production in patients with gastrointestinal cancer. *Br J Surg* **73**: 483–486

Morrissey P, Bressler L, Park L, Alpert A and Gillis S (1987) Granulocyte–macrophage colon-stimulating factor augments the primary antibody response by enhancing the function of antigen presenting cells. *J Immunol* **139**: 1113–1119

Morton BA, O'Connor-Tressel M, Beatty BG, Shively JE and Beatty JD (1988) Artefactual CEA elevation due to human anti-mouse antibodies. *Arch Surg* **123**: 1242–1246

Moss B and Flexner C (1987) Vaccinia virus expression vectors. *Ann Rev Immunol* **5**: 305–324

Moss B, Smith GL, Gerin JL and Purcell RH (1984) Live recombinant vaccinia virus protects chimpanzees against Hepatitis B. *Nature* **311**: 67–69

Muraro R, Wunderlich D and Thor A (1985) Definition by monoclonal antibodies of a repertoire of epitopes on CEA differentially expressed in human colon carcinomas versus normal adult tissues. *Cancer Res* **45**: 5769–5780

Nap M, Mollgard K, Burtin P and Fleuren GJ (1988) Immunohistochemistry of carcinoembryonic antigen in the embryo, fetus and adult. *Tumor Biol* **9**: 145–153

Nieroda C, Milenic DE, Carrasquillo JA, Schlom J and Greiner JW (1995) Improved tumour radioimmunodetection using a single-chain Fv and interferon-γ: potential clinical appilcations for radioimmunoguided surgery and γ-scanning. *Cancer Res* **55**: 2858–2865

Nijman HN, Houbiers JG, Vierbom MP, van der Burg SH, Drijfhout JW, D'Amaro J, Kenemans P, Melief CJ and Kast WM (1993) Identification of peptide sequences that potentially trigger HLA-A2.1 restricted cytotoxic T lymphocytes. *Eur J Immunol* **23**: 1215–1219

Nossal GJV (1983) Cellular mechanisms of immunological tolerance. *Ann Rev Immunol* **1**: 33–62

Nossal GJV and Pike BL (1980) Clonal anergy: persistence in tolerant mice of antigen binding B lymphocytes incapable of responding to antigen or mitogen. *Proc Natl Acad Sci USA* **77**: 1602–1606

Oosterwijk E, Warnaar SO, Zwartendijk J, Van der Velde EA, Fleuren GJ and Cornelisse CJ (1988) Relationship between DNA ploidy, antigen expression and survival in renal cell carcinoma. *Int J Cancer* **42**: 703–708

Osborne M, Wong GY, Asina S, Old LJ, Cote RJ and Rosen PP (1991) Sensitivity of immunocytochemical detection of breast cancer cells in human bone marrow. *Cancer Res* **51**: 2706–2709

Pertmer TM, Eisenbraun MD and McCabe D (1997) Gene gun based nucleic acid immunization: elicitation of humoral and cytotoxic T lymphocyte responses following epidermal delivery of nanogram quantities of DNA. *Vaccine* **13**: 1427–1430

Pressman D, Chu TM and Grossberg AL (1980) Carcinoembryonic antigen binding immunoglobulin isolated from normal human serum by affinity chromatography. *Transpl Proc* **12**: 195–197

Raganhammar P, Fagerberg J, Frodin J-E, Hjelm A-L, Lindemalm C, Magnusson I, Masucci G and Mellstedt H (1993) Effect of monoclonal antibody 17-1A and GM-CSF in patients with advanced colorectal carcinoma – long-lasting complete remissions can be induced. *Int J Cancer* **53**: 751–758

Raganhammar P, Fagerberg J, Frodin J-E, Wersall P, Hansson L-O and Mellstedt H (1995) Granulocyte/macrophage colony-stimulating factor augments the induction of antibodies, especially anti-idiotypic antibodies, to therapeutic monoclonal antibodies. *Cancer Immunol Immunother* **40**: 367–375

Raychaudhuri S, Kang C-Y, Kaveri S-V, Kieber-Emmons T and Kohler H (1990) Tumour idiotype vaccines: VII. Analysis and correlation of structural, idiotypic and biologic properties of protective and non-protective $Ab_2$. *J Immunol* **145**: 760–767

Richman P and Bodmer WF (1987) Monoclonal antibodies to human colorectal epithelum: markers for differentiation and tumour characterization. *Int J Cancer* **39**: 317–328

Riethmuller G and Johnson GP (1992) Monoclonal antibodies in the detection and therapy of micrometastatic epithelial cancers. *Curr Opin Immunol* **4**: 647–655

Riethmuller G, Schneider-Gadicke E, Schlimok W, Raab R, Hooken K, Gruber R and the German Cancer Aid 17-1A Study Group (1994) Randomized trial of monoclonal antibody for adjuvant therapy of resected Dukes' C colorectal carcinoma. *Lancet* **343**: 1177–1183

Robins RA, Denton GWL, Austin EB, Hardcastle JD and Durrant LG (1991*a*) Anti-tumour immune responses and interleukin-2 production in colorectal cancer patients by immunization with human monoclonal anti-idiotypic antibody. *Cancer Res* **51**: 5425–5429

Robins RA, Denton GWL, Austin EB, Hardcastle JD and Durrant LG (1991*b*) Anti-tumour immune responses and interleukin-2 production in colorectal cancer patients by immunization with human monoclonal anti-idiotypic antibody. *Cancer Res* **51**: 5425–5429

Rodenburg CJ, Cornelisse CJ, Heintz APM, Hermans J and Fleuren GJ (1987) Tumour ploidy as a major prognostic factor in advanced ovarian cancer. *Cancer* **59**: 317–323

Rosa FM and Fellous M (1988) Regulation of HLA-DR gene by IFN-γ: transcriptional and post-transcriptional control. *J Immunol* **140**: 1660–1664

Rosenberg SA, Lotze MT, Yang JC, Aebersold PM, Linehan WM, Seip CA and White DE (1989) Experience with the use of high-dose interleukin-2 in the treatment of 652 cancer patients. *Ann Surg* **210**: 474–485

Schlimok G, Funke I, Bock B, Schweiberer B, Witte J and Riethmuller G (1990) Epithelial tumour cells in the bone marrow of patients with colorectal cancer: immunocytochemical detection, phenotypic characterization and prognosis significance. *J Clin Oncol* **8**: 831–837

Shortman K (1992) Cellular aspects of early T cell development. *Curr Opin Immunol* **4**: 140–146

Sidransky D, Tokino T, Hamilton SR, Kinzler KW, Levin B, Frost P and Vogelstein B (1992) Identification of *ras* oncogene mutations in the stool of patients with curable colorectal tumours. *Science* **256**: 102–105

Sieff CA, Emerson SG, Donahue RE, Nathan DG, Wang EA, Wong GG and Clark SC (1985) Human recombinant granulocyte–macrophage colony-stimulating factor: a multilineage haematopoietin. *Science* **230**: 1171–1173

Slingluff CL, Cox AL, Henderson RA, Hunt DF and Engelhard VH (1993) Recognition of human melanoma cells by HLA-A2.1 restricted cytotoxic T lymphocytes is mediated by at least six shared peptide epitopes. *J Immunol* **150**: 2955–2963

Slovin SV, Lackman RD, Ferrone S, Kiely PE and Mastrangelo MJ (1986) Cellular immune response to human sarcomas: cytotoxic T cell clones reactive with autologous sarcomas. *J Immunol* **137**: 3042–3048

Sorokin JJ, Kupchick HZ and Zamchek N (1973) Carcinoembryonic antigen in colon cancer: absence in perchloric acid precipitates in plasma. *J Natl Cancer Inst* **51**: 1081–1083

Staab HJ, Anderer FA, Stumpf E and Fischer R (1980) Are circulating CEA immune complexes a prognostic marker in patients with carcinoma of the gastrointestinal tract? *Br J Cancer* **42**: 26–33

Steplewski Z, Lubeck MD and Koprowski H (1983) Human macrophages armed with murine $IgG_{2A}$ anti-tumour immunoglobulins destroy human cancer cells. *Science* **221**: 865–876

© Cancer Research Campaign 1998

Steplewski Z, Herlyn D, Lubeck M, Kimoto Y, Herlyn M and Koprowski H (1986) Mechanisms of tumour growth inhibition. *Hybridoma* **5** (suppl. 1): S59–S62

Thompson J and Zimmerman W (1988) The carcinoembryonic gene family: structure, expression and evolution. *Tumour Biol* **9**: 63–83

Thompson JA, Grunert F and Zimmerman W (1991) Carcinoembryonic antigen gene family: molecular biology and clinical perspectives. *J Clin Lab Anal* **5**: 344–366

Trauth BC, Klas C, Peters AMJ, Matzku S, Moller P, Falk W, Debatin KM and Krummer PH (1989) Monoclonal antibody mediated tumour regression by induction of apoptosis. *Science* **245**: 301–305

Tsang KY, Zaremba S, Nieroda CA, Zhu MZ, Hamilton JM and Schlom J (1995) Generation of human cytotoxic T cells specific for human carcinoembryonic antigen epitopes from patients immunized with recombinant vaccinia-CEA vaccine. *J Natl Cancer Inst* **87**: 982–990

Tynan K, Olsen A, Trask B, de Jong P, Thompson J, Zimmerman W, Carrano A and Mohrenweiser H (1992) Assembly and analysis of cosmid contigs in the CEA-gene family region of human chromosome 19. *Nucleic Acids Res* **20**: 1629–1636

Ura Y, Ochi Y, Hamazu M, Ishida M, Nakajima K and Watanabe T (1985) Studies on circulating anitbody against carcinoembryonic antigen (CEA) and CEA-like antigen in cancer patients. *Cancer Lett* **25**: 283–285

Wadhwa M, Bird C, Fagerberg J, Gaines-Das R, Raganhammar P and Mellstedt H (1996) Production of neutralizing granulocyte–macrophage colony stimulating factor (GM-CSF) antibodies in carcinoma patients following GM-CSF combination therapy. *Clin Exp Immunol* **104**: 351–358

Wang B, Boyer J, Srikantan V, Coney L, Carrano R, Phan C, Merva M, Dang K, Agadjanan M and Gilbert L (1993) DNA inoculation induces neutralizing immune responses against human immunodeficiency virus type I in mice and non human primates. *DNA Cell Biol* **12**: 799–805

Williams AF and Barclay AN (1988) The immunoglobulin superfamily – domains for cell surface recognition. *Annu Rev Immunol* **6**: 381–405

Winter G and Milstein C (1991) Man-made antibodies. *Nature* **349**: 293–299

Wolfel T, Schneider J, Meyer zum Buschenfelde K-H, Rammensee H-G, Rotzchke O and Falk K (1994) Isolation of naturally processed peptides recognized by cytolytic T lymphocytes (CTL) on human melanoma cells in association with HLA-A2. *Int J Cancer* **57**: 413–418

Wolff JA, Malone RW, Williams P, Chong W, Acsadi G, Jani A and Felgner PL (1990) Direct gene transfer into mouse muscle in vivo. *Science* **247**: 1465–1468

Wolff JA, Ludike JJ, Acsadi G, Williams P and Jani A (1992) Long term persistence of plasmid DNA and foreign gene expression in mouse muscle. *Hum Molec Genet* **1**: 363–369

Wyllie AH, Kerr JFR and Currie AR (1980) Cell death: the significance of apoptosis. *Int Rev Cytol* **68**: 251–306

Yang N-S, Burkholder J, Roberts B, Montinell B and McCabe D (1990) In vivo and in vitro gene transfer to mammalian somatic cells by particle bombardment. *Proc Natl Acad Sci USA* **87**: 9568–9572

Zhou X, Berglund P, Zhao H, Liljestrom P and Jondal M (1995) Generation of cytotoxic and humoral immune responses by nonreplicative recombinant Semiliki Forest virus. *Proc Natl Acad Sci USA* **92**: 3009–3013

© Cancer Research Campaign 1998


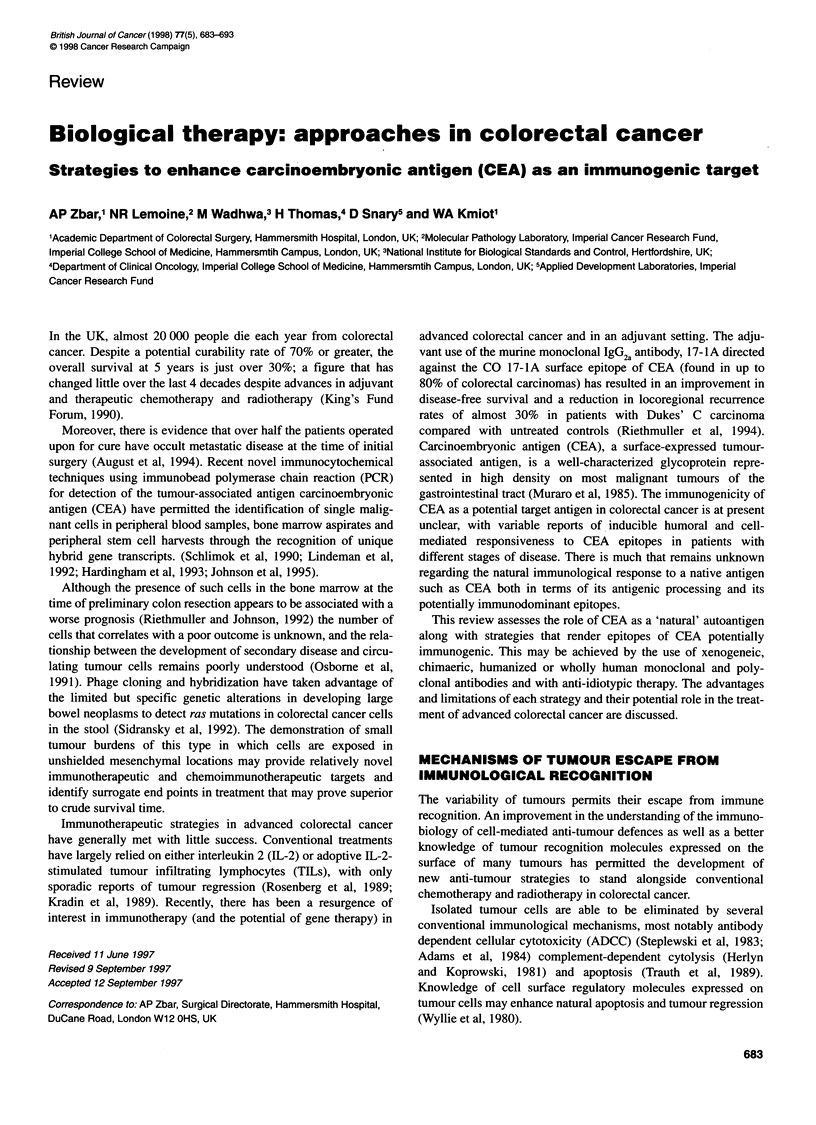

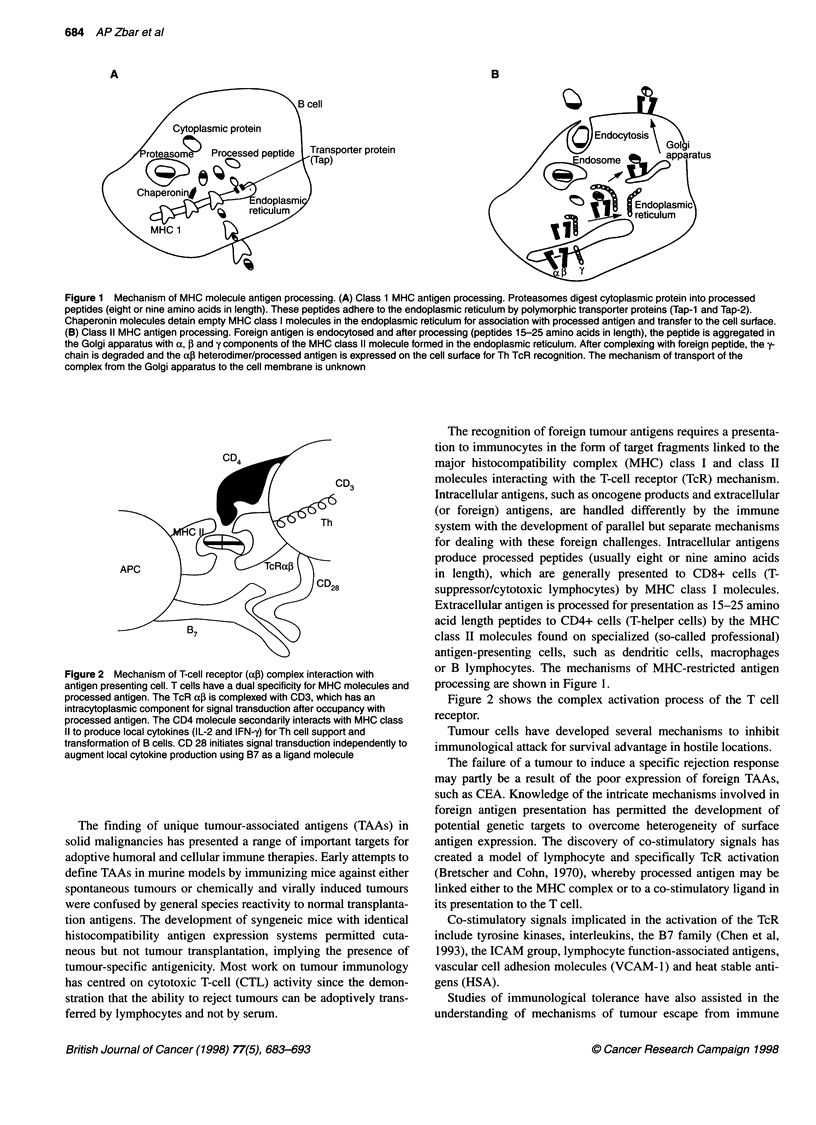

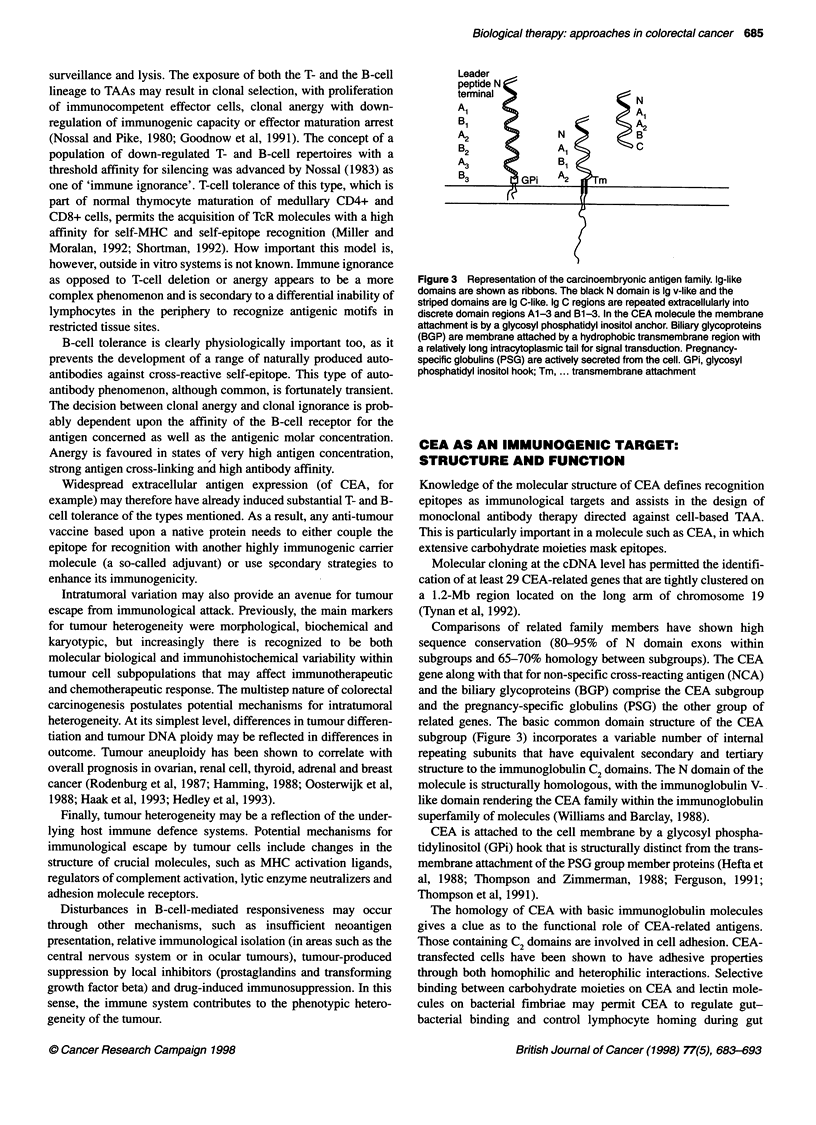

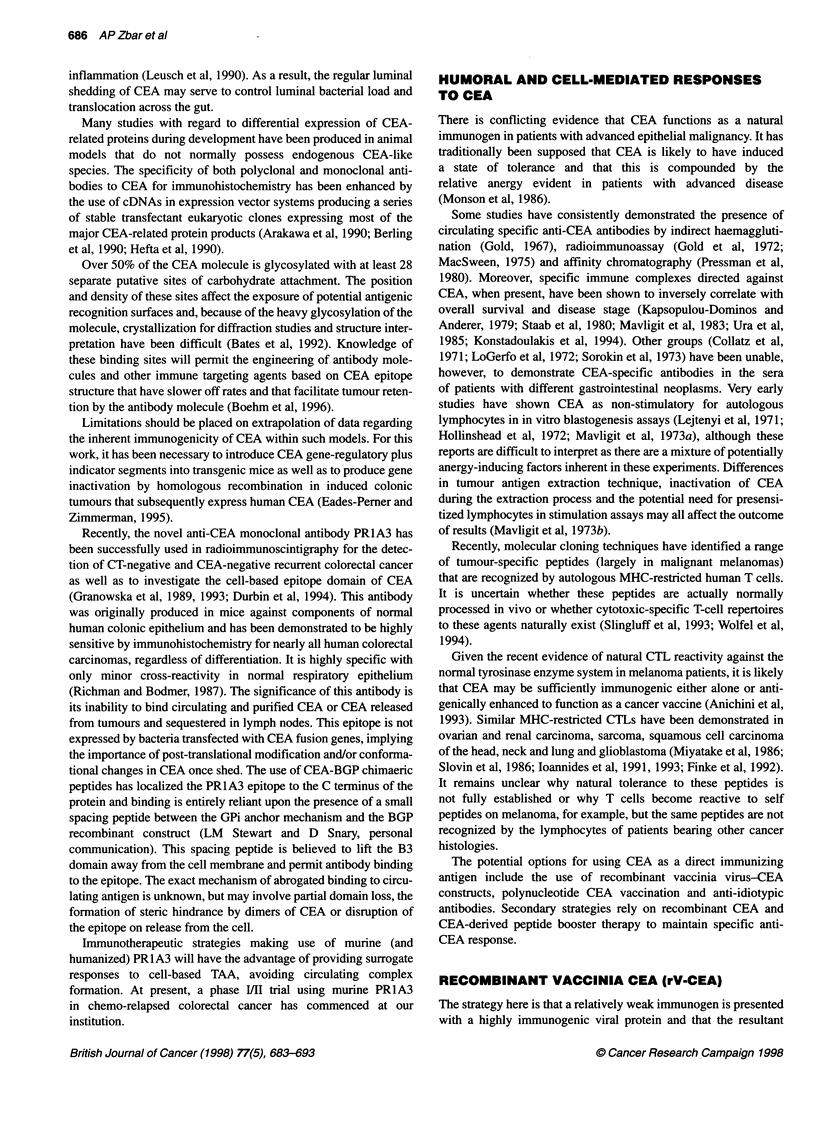

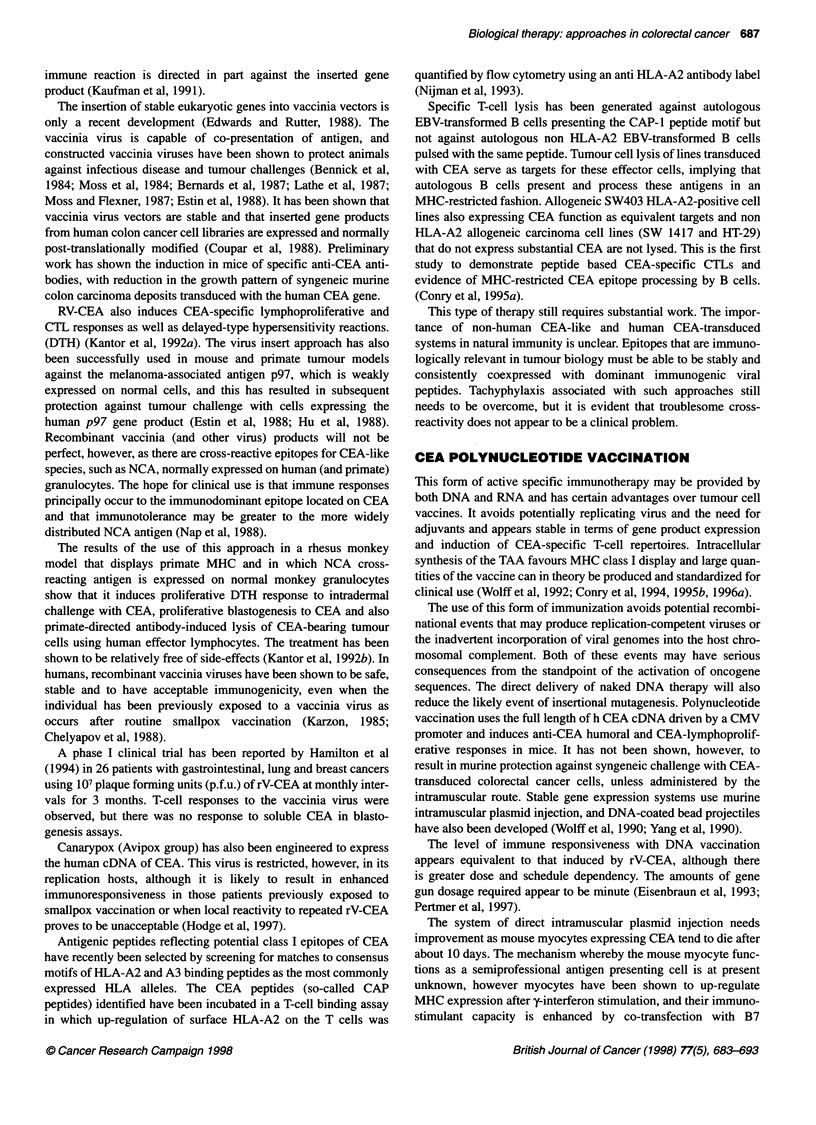

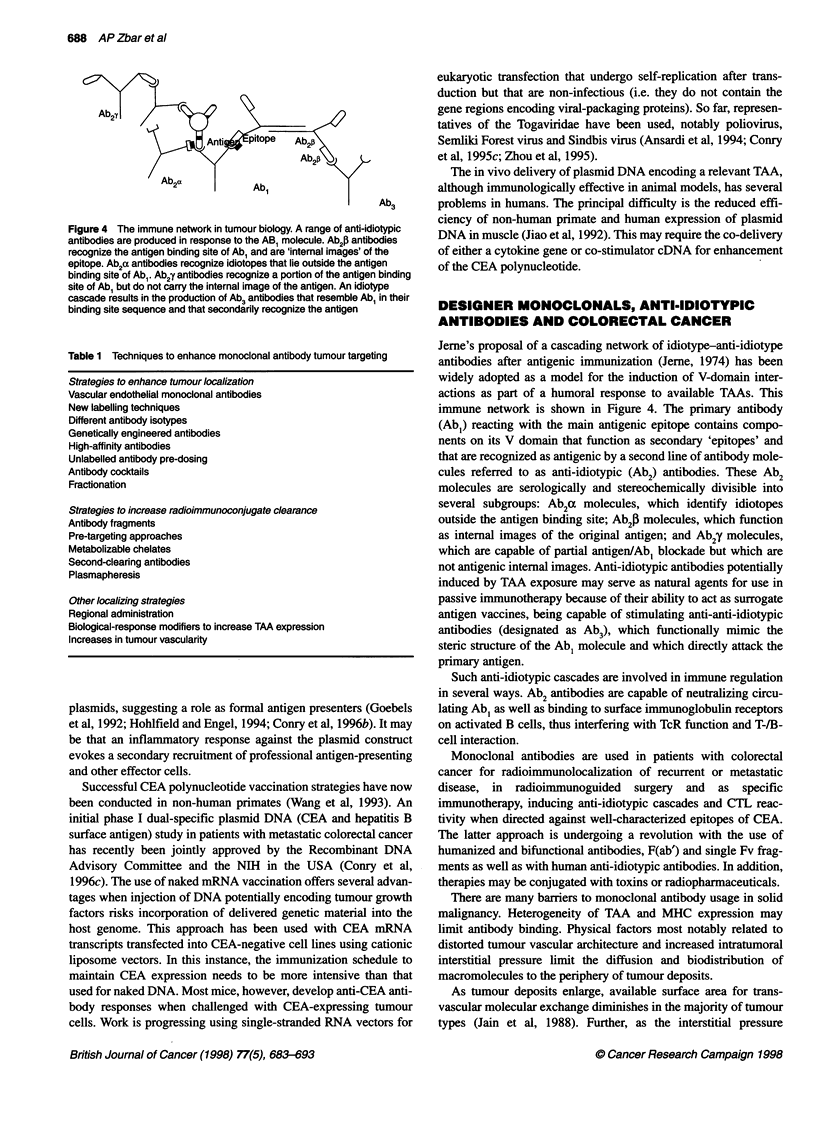

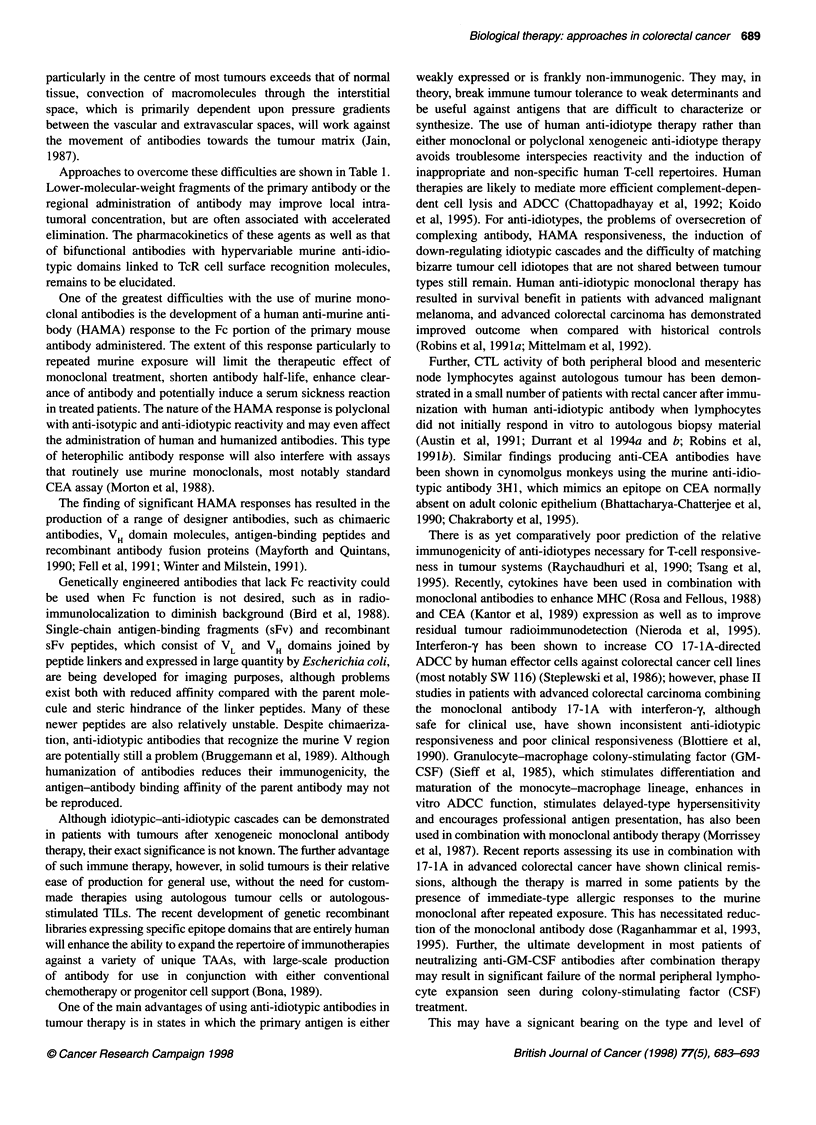

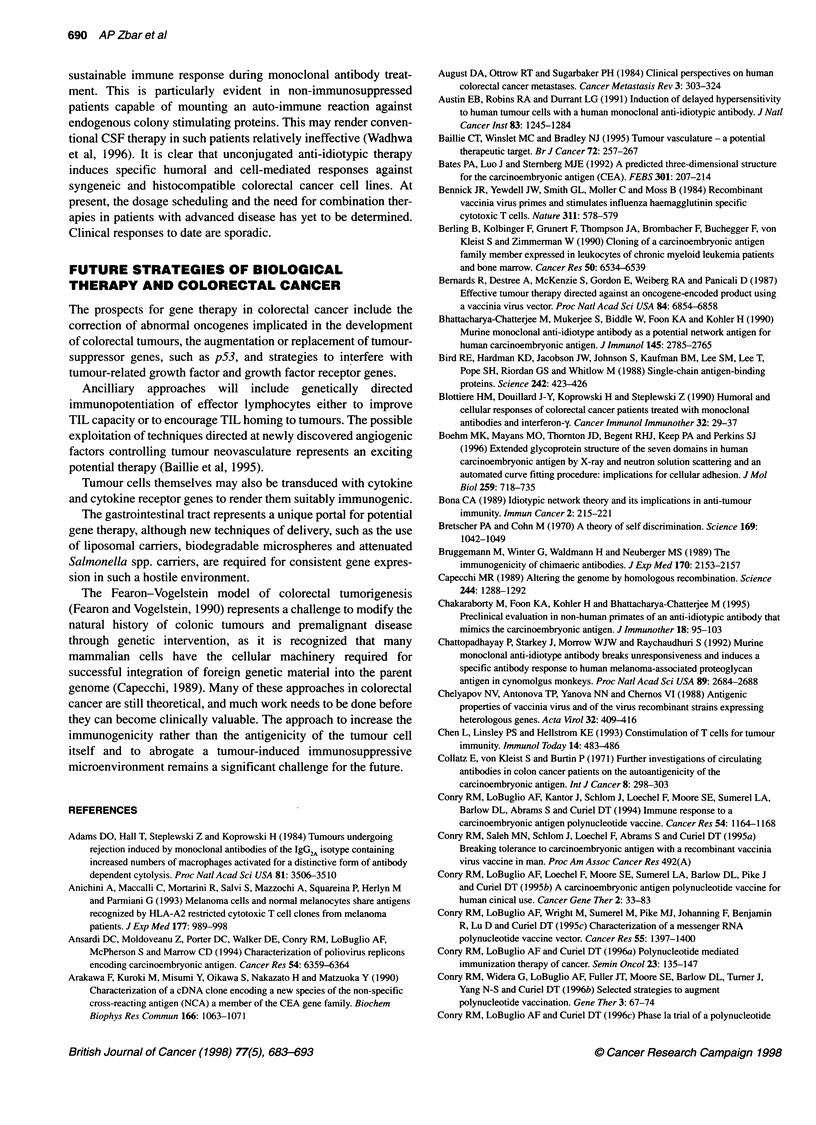

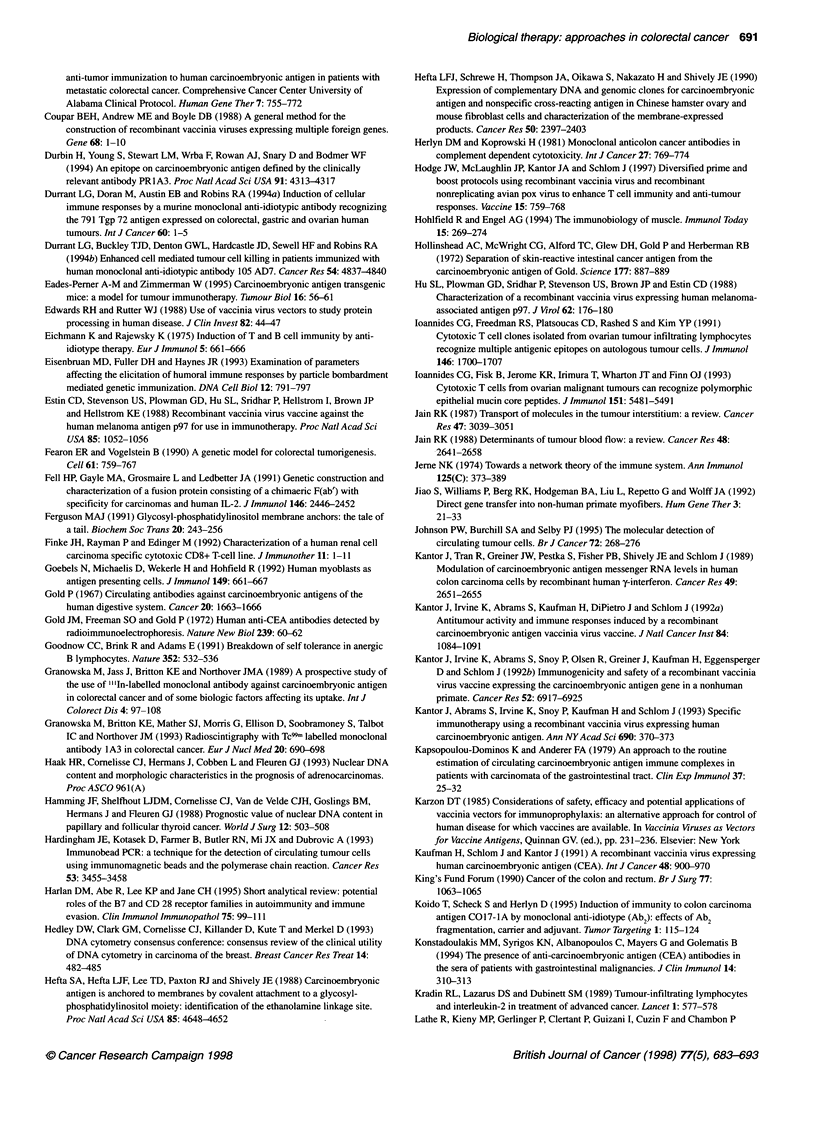

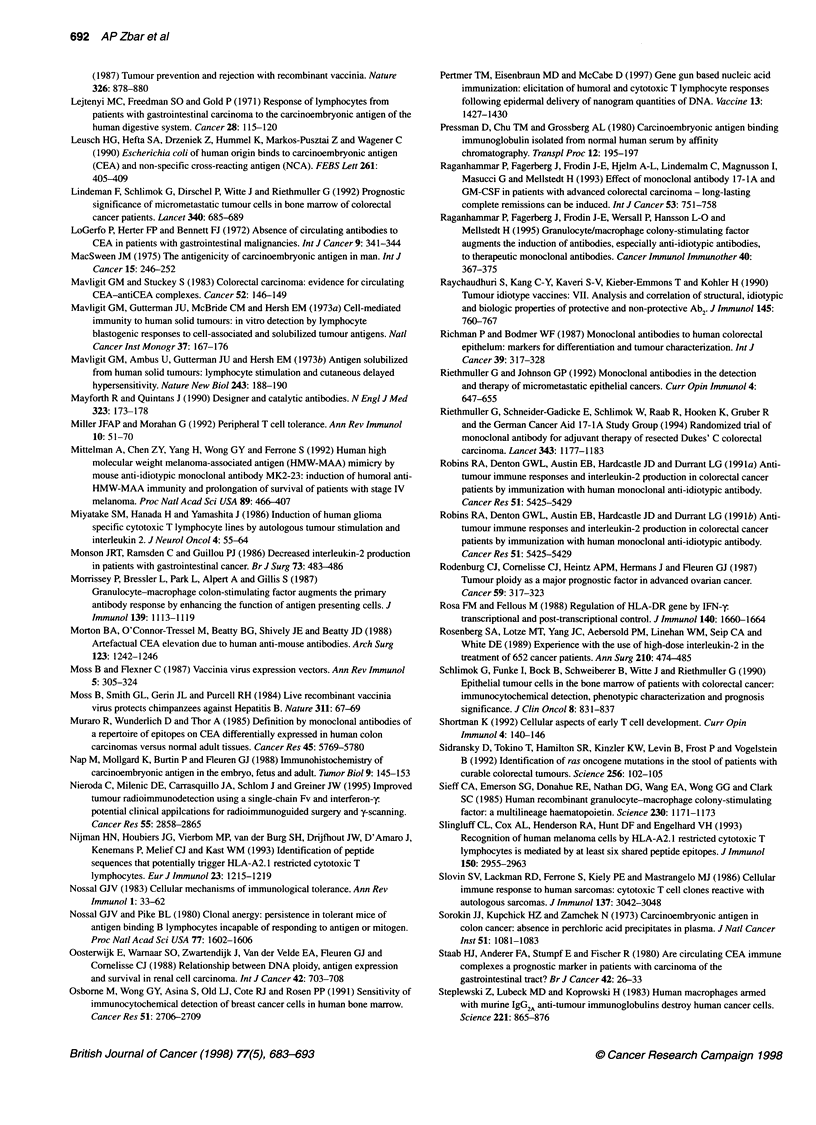

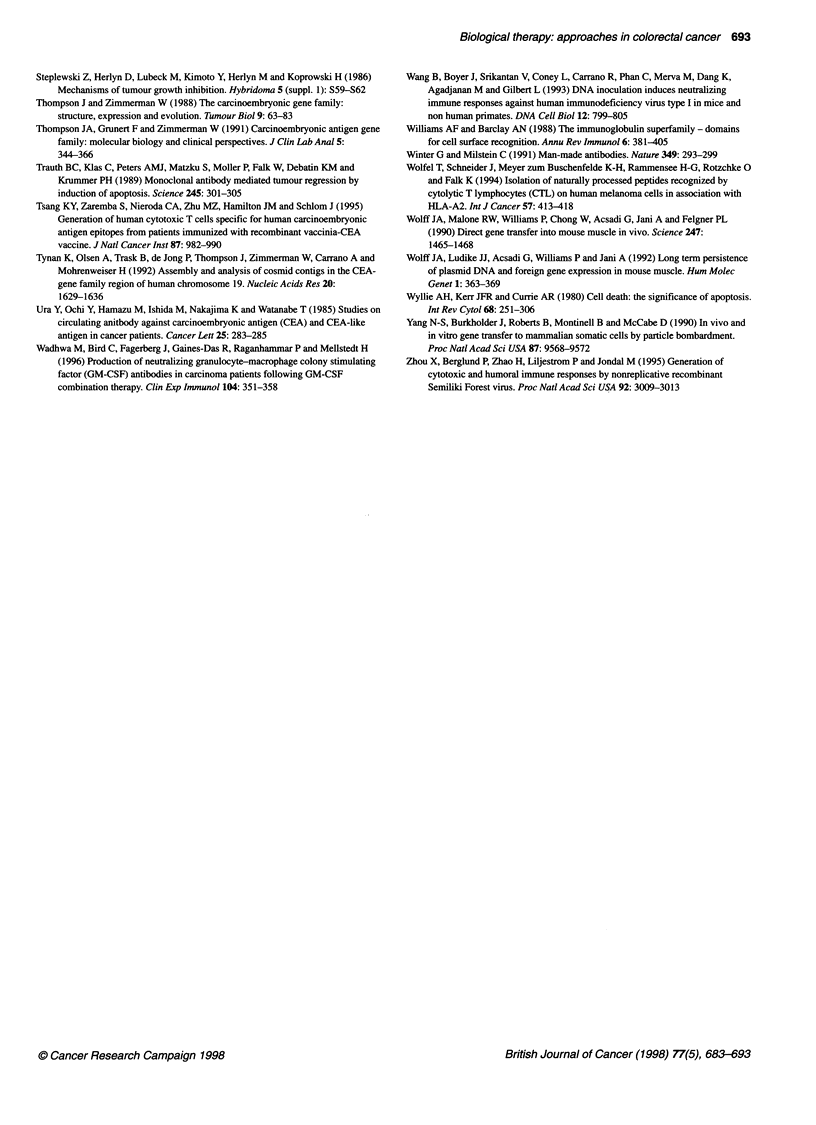


## References

[OCR_00975] Adams D. O., Hall T., Steplewski Z., Koprowski H. (1984). Tumors undergoing rejection induced by monoclonal antibodies of the IgG2a isotype contain increased numbers of macrophages activated for a distinctive form of antibody-dependent cytolysis.. Proc Natl Acad Sci U S A.

[OCR_00982] Anichini A., Maccalli C., Mortarini R., Salvi S., Mazzocchi A., Squarcina P., Herlyn M., Parmiani G. (1993). Melanoma cells and normal melanocytes share antigens recognized by HLA-A2-restricted cytotoxic T cell clones from melanoma patients.. J Exp Med.

[OCR_00988] Ansardi D. C., Moldoveanu Z., Porter D. C., Walker D. E., Conry R. M., LoBuglio A. F., McPherson S., Morrow C. D. (1994). Characterization of poliovirus replicons encoding carcinoembryonic antigen.. Cancer Res.

[OCR_00993] Arakawa F., Kuroki M., Misumi Y., Oikawa S., Nakazato H., Matsuoka Y. (1990). Characterization of a cDNA clone encoding a new species of the nonspecific cross-reacting antigen (NCA), a member of the CEA gene family.. Biochem Biophys Res Commun.

[OCR_00999] August D. A., Ottow R. T., Sugarbaker P. H. (1984). Clinical perspective of human colorectal cancer metastasis.. Cancer Metastasis Rev.

[OCR_01003] Austin E. B., Robins R. A., Baldwin R. W., Durrant L. G. (1991). Induction of delayed hypersensitivity to human tumor cells with a human monoclonal anti-idiotypic antibody.. J Natl Cancer Inst.

[OCR_01008] Baillie C. T., Winslet M. C., Bradley N. J. (1995). Tumour vasculature--a potential therapeutic target.. Br J Cancer.

[OCR_01012] Bates P. A., Luo J., Sternberg M. J. (1992). A predicted three-dimensional structure for the carcinoembryonic antigen (CEA).. FEBS Lett.

[OCR_01016] Bennink J. R., Yewdell J. W., Smith G. L., Moller C., Moss B. (1984). Recombinant vaccinia virus primes and stimulates influenza haemagglutinin-specific cytotoxic T cells.. Nature.

[OCR_01021] Berling B., Kolbinger F., Grunert F., Thompson J. A., Brombacher F., Buchegger F., von Kleist S., Zimmermann W. (1990). Cloning of a carcinoembryonic antigen gene family member expressed in leukocytes of chronic myeloid leukemia patients and bone marrow.. Cancer Res.

[OCR_01028] Bernards R., Destree A., McKenzie S., Gordon E., Weinberg R. A., Panicali D. (1987). Effective tumor immunotherapy directed against an oncogene-encoded product using a vaccinia virus vector.. Proc Natl Acad Sci U S A.

[OCR_01033] Bhattacharya-Chatterjee M., Mukerjee S., Biddle W., Foon K. A., Köhler H. (1990). Murine monoclonal anti-idiotype antibody as a potential network antigen for human carcinoembryonic antigen.. J Immunol.

[OCR_01038] Bird R. E., Hardman K. D., Jacobson J. W., Johnson S., Kaufman B. M., Lee S. M., Lee T., Pope S. H., Riordan G. S., Whitlow M. (1988). Single-chain antigen-binding proteins.. Science.

[OCR_01043] Blottiere H. M., Douillard J. Y., Koprowski H., Steplewski Z. (1990). Humoral and cellular responses of colorectal cancer patients treated with monoclonal antibodies and interferon gamma.. Cancer Immunol Immunother.

[OCR_01048] Boehm M. K., Mayans M. O., Thornton J. D., Begent R. H., Keep P. A., Perkins S. J. (1996). Extended glycoprotein structure of the seven domains in human carcinoembryonic antigen by X-ray and neutron solution scattering and an automated curve fitting procedure: implications for cellular adhesion.. J Mol Biol.

[OCR_01057] Bona C. A. (1989). Idiotype network theory and its implications in anti-tumor immunity.. Prog Clin Biol Res.

[OCR_01061] Bretscher P., Cohn M. (1970). A theory of self-nonself discrimination.. Science.

[OCR_01065] Brüggemann M., Winter G., Waldmann H., Neuberger M. S. (1989). The immunogenicity of chimeric antibodies.. J Exp Med.

[OCR_01069] Capecchi M. R. (1989). Altering the genome by homologous recombination.. Science.

[OCR_01073] Chakraborty M., Foon K. A., Kohler H., Bhattacharya-Chatterjee M. (1995). Preclinical evaluation in nonhuman primates of an anti-idiotypic antibody that mimicks the carcinoembryonic antigen.. J Immunother Emphasis Tumor Immunol.

[OCR_01078] Chattopadhyay P., Starkey J., Morrow W. J., Raychaudhuri S. (1992). Murine monoclonal anti-idiotope antibody breaks unresponsiveness and induces a specific antibody response to human melanoma-associated proteoglycan antigen in cynomolgus monkeys.. Proc Natl Acad Sci U S A.

[OCR_01084] Chelyapov N. V., Antonova T. P., Yanova N. N., Chernos V. I. (1988). Antigenic properties of vaccinia virus and of the virus recombinant strains expressing heterologous genes.. Acta Virol.

[OCR_01089] Chen L., Linsley P. S., Hellström K. E. (1993). Costimulation of T cells for tumor immunity.. Immunol Today.

[OCR_01093] Collatz E., Von Kleist S., Burtin P. (1971). Further investigations of circulating antibodies in colon cancer patients: on the autoantigenicity of the carcinoembryonic antigen.. Int J Cancer.

[OCR_01118] Conry R. M., LoBuglio A. F., Curiel D. T. (1996). Polynucleotide-mediated immunization therapy of cancer.. Semin Oncol.

[OCR_01098] Conry R. M., LoBuglio A. F., Kantor J., Schlom J., Loechel F., Moore S. E., Sumerel L. A., Barlow D. L., Abrams S., Curiel D. T. (1994). Immune response to a carcinoembryonic antigen polynucleotide vaccine.. Cancer Res.

[OCR_01108] Conry R. M., LoBuglio A. F., Loechel F., Moore S. E., Sumerel L. A., Barlow D. L., Pike J., Curiel D. T. (1995). A carcinoembryonic antigen polynucleotide vaccine for human clinical use.. Cancer Gene Ther.

[OCR_01113] Conry R. M., LoBuglio A. F., Wright M., Sumerel L., Pike M. J., Johanning F., Benjamin R., Lu D., Curiel D. T. (1995). Characterization of a messenger RNA polynucleotide vaccine vector.. Cancer Res.

[OCR_01122] Conry R. M., Widera G., LoBuglio A. F., Fuller J. T., Moore S. E., Barlow D. L., Turner J., Yang N. S., Curiel D. T. (1996). Selected strategies to augment polynucleotide immunization.. Gene Ther.

[OCR_01139] Coupar B. E., Andrew M. E., Boyle D. B. (1988). A general method for the construction of recombinant vaccinia viruses expressing multiple foreign genes.. Gene.

[OCR_01144] Durbin H., Young S., Stewart L. M., Wrba F., Rowan A. J., Snary D., Bodmer W. F. (1994). An epitope on carcinoembryonic antigen defined by the clinically relevant antibody PR1A3.. Proc Natl Acad Sci U S A.

[OCR_01155] Durrant L. G., Buckley T. J., Denton G. W., Hardcastle J. D., Sewell H. F., Robins R. A. (1994). Enhanced cell-mediated tumor killing in patients immunized with human monoclonal antiidiotypic antibody 105AD7.. Cancer Res.

[OCR_01160] Eades-Perner A. M., Zimmermann W. (1995). Carcinoembryonic antigen-transgenic mice: a model for tumor immunotherapy.. Tumour Biol.

[OCR_01164] Edwards R. H., Rutter W. J. (1988). Use of vaccinia virus vectors to study protein processing in human disease. Normal nerve growth factor processing and secretion in cultured fibroblasts from patients with familial dysautonomia.. J Clin Invest.

[OCR_01168] Eichmann K., Rajewsky K. (1975). Induction of T and B cell immunity by anti-idiotypic antibody.. Eur J Immunol.

[OCR_01172] Eisenbraun M. D., Fuller D. H., Haynes J. R. (1993). Examination of parameters affecting the elicitation of humoral immune responses by particle bombardment-mediated genetic immunization.. DNA Cell Biol.

[OCR_01177] Estin C. D., Stevenson U. S., Plowman G. D., Hu S. L., Sridhar P., Hellström I., Brown J. P., Hellström K. E. (1988). Recombinant vaccinia virus vaccine against the human melanoma antigen p97 for use in immunotherapy.. Proc Natl Acad Sci U S A.

[OCR_01184] Fearon E. R., Vogelstein B. (1990). A genetic model for colorectal tumorigenesis.. Cell.

[OCR_01188] Fell H. P., Gayle M. A., Grosmaire L., Ledbetter J. A. (1991). Genetic construction and characterization of a fusion protein consisting of a chimeric F(ab') with specificity for carcinomas and human IL-2.. J Immunol.

[OCR_01193] Ferguson M. A. (1992). Colworth Medal Lecture. Glycosyl-phosphatidylinositol membrane anchors: the tale of a tail.. Biochem Soc Trans.

[OCR_01197] Finke J. H., Rayman P., Edinger M., Tubbs R. R., Stanley J., Klein E., Bukowski R. (1992). Characterization of a human renal cell carcinoma specific cytotoxic CD8+ T cell line.. J Immunother (1991).

[OCR_01201] Goebels N., Michaelis D., Wekerle H., Hohlfeld R. (1992). Human myoblasts as antigen-presenting cells.. J Immunol.

[OCR_01209] Gold J. M., Freedman S. O., Gold P. (1972). Human anti-CEA antibodies detected by radioimmunoelectrophoresis.. Nat New Biol.

[OCR_01213] Goodnow C. C., Brink R., Adams E. (1991). Breakdown of self-tolerance in anergic B lymphocytes.. Nature.

[OCR_01223] Granowska M., Britton K. E., Mather S. J., Morris G., Ellison D., Soobramoney S., Talbot I. C., Northover J. M. (1993). Radioimmunoscintigraphy with technetium-99m labelled monoclonal antibody, 1A3, in colorectal cancer.. Eur J Nucl Med.

[OCR_01217] Granowska M., Jass J. R., Britton K. E., Northover J. M. (1989). A prospective study of the use of 111In-labelled monoclonal antibody against carcino-embryonic antigen in colorectal cancer and of some biological factors affecting its uptake.. Int J Colorectal Dis.

[OCR_01233] Hamming J. F., Schelfhout L. J., Cornelisse C. J., van de Velde C. J., Goslings B. M., Hermans J., Fleuren G. J. (1988). Prognostic value of nuclear DNA content in papillary and follicular thyroid cancer.. World J Surg.

[OCR_01238] Hardingham J. E., Kotasek D., Farmer B., Butler R. N., Mi J. X., Sage R. E., Dobrovic A. (1993). Immunobead-PCR: a technique for the detection of circulating tumor cells using immunomagnetic beads and the polymerase chain reaction.. Cancer Res.

[OCR_01245] Harlan D. M., Abe R., Lee K. P., June C. H. (1995). Potential roles of the B7 and CD28 receptor families in autoimmunity and immune evasion.. Clin Immunol Immunopathol.

[OCR_01250] Hedley D. W., Clark G. M., Cornelisse C. J., Killander D., Kute T., Merkel D. (1993). Consensus review of the clinical utility of DNA cytometry in carcinoma of the breast. Report of the DNA Cytometry Consensus Conference.. Cytometry.

[OCR_01263] Hefta L. J., Schrewe H., Thompson J. A., Oikawa S., Nakazato H., Shively J. E. (1990). Expression of complementary DNA and genomic clones for carcinoembryonic antigen and nonspecific cross-reacting antigen in Chinese hamster ovary and mouse fibroblast cells and characterization of the membrane-expressed products.. Cancer Res.

[OCR_01256] Hefta S. A., Hefta L. J., Lee T. D., Paxton R. J., Shively J. E. (1988). Carcinoembryonic antigen is anchored to membranes by covalent attachment to a glycosylphosphatidylinositol moiety: identification of the ethanolamine linkage site.. Proc Natl Acad Sci U S A.

[OCR_01270] Herlyn D. M., Koprowski H. (1981). Monoclonal anticolon carcinoma antibodies in complement-dependent cytotoxicity.. Int J Cancer.

[OCR_01274] Hodge J. W., McLaughlin J. P., Kantor J. A., Schlom J. (1997). Diversified prime and boost protocols using recombinant vaccinia virus and recombinant non-replicating avian pox virus to enhance T-cell immunity and antitumor responses.. Vaccine.

[OCR_01281] Hohlfeld R., Engel A. G. (1994). The immunobiology of muscle.. Immunol Today.

[OCR_01285] Hollinshead A. C., McWright C. G., Alford TC GLEW D. H., Gold P., Herbeman R. B. (1972). Separation of skin reactive intestinal cancer antigen from the carcinoembryonic antigen of Gold.. Science.

[OCR_01290] Hu S. L., Plowman G. D., Sridhar P., Stevenson U. S., Brown J. P., Estin C. D. (1988). Characterization of a recombinant vaccinia virus expressing human melanoma-associated antigen p97.. J Virol.

[OCR_01295] Ioannides C. G., Freedman R. S., Platsoucas C. D., Rashed S., Kim Y. P. (1991). Cytotoxic T cell clones isolated from ovarian tumor-infiltrating lymphocytes recognize multiple antigenic epitopes on autologous tumor cells.. J Immunol.

[OCR_01310] Jain R. K. (1988). Determinants of tumor blood flow: a review.. Cancer Res.

[OCR_01306] Jain R. K. (1987). Transport of molecules in the tumor interstitium: a review.. Cancer Res.

[OCR_01314] Jerne N. K. (1974). Towards a network theory of the immune system.. Ann Immunol (Paris).

[OCR_01318] Jiao S., Williams P., Berg R. K., Hodgeman B. A., Liu L., Repetto G., Wolff J. A. (1992). Direct gene transfer into nonhuman primate myofibers in vivo.. Hum Gene Ther.

[OCR_01323] Johnson P. W., Burchill S. A., Selby P. J. (1995). The molecular detection of circulating tumour cells.. Br J Cancer.

[OCR_01346] Kantor J., Abrams S., Irvine K., Snoy P., Kaufman H., Schlom J. (1993). Specific immunotherapy using a recombinant vaccinia virus expressing human carcinoembryonic antigen.. Ann N Y Acad Sci.

[OCR_01333] Kantor J., Irvine K., Abrams S., Kaufman H., DiPietro J., Schlom J. (1992). Antitumor activity and immune responses induced by a recombinant carcinoembryonic antigen-vaccinia virus vaccine.. J Natl Cancer Inst.

[OCR_01340] Kantor J., Irvine K., Abrams S., Snoy P., Olsen R., Greiner J., Kaufman H., Eggensperger D., Schlom J. (1992). Immunogenicity and safety of a recombinant vaccinia virus vaccine expressing the carcinoembryonic antigen gene in a nonhuman primate.. Cancer Res.

[OCR_01327] Kantor J., Tran R., Greiner J., Pestka S., Fisher P. B., Shively J. E., Schlom J. (1989). Modulation of carcinoembryonic antigen messenger RNA levels in human colon carcinoma cells by recombinant human gamma-interferon.. Cancer Res.

[OCR_01351] Kapsopoulou-Dominos K., Anderer F. A. (1979). An approach to the routine estimation of circulating carcinoembryonic antigen immune complexes in patients with carcinomata of the gastrointestinal tract.. Clin Exp Immunol.

[OCR_01364] Kaufman H., Schlom J., Kantor J. (1991). A recombinant vaccinia virus expressing human carcinoembryonic antigen (CEA).. Int J Cancer.

[OCR_01376] Konstadoulakis M. M., Syrigos K. N., Albanopoulos C., Mayers G., Golematis B. (1994). The presence of anti-carcinoembryonic antigen (CEA) antibodies in the sera of patients with gastrointestinal malignancies.. J Clin Immunol.

[OCR_01382] Kradin R. L., Kurnick J. T., Lazarus D. S., Preffer F. I., Dubinett S. M., Pinto C. E., Gifford J., Davidson E., Grove B., Callahan R. J. (1989). Tumour-infiltrating lymphocytes and interleukin-2 in treatment of advanced cancer.. Lancet.

[OCR_01397] Lejtenyi M. C., Freedman S. O., Gold P. (1971). Response of lymphocytes from patients with gastrointestinal cancer to the carcinoembryonic antigen of the human digestive system.. Cancer.

[OCR_01402] Leusch H. G., Hefta S. A., Drzeniek Z., Hummel K., Markos-Pusztai Z., Wagener C. (1990). Escherichia coli of human origin binds to carcinoembryonic antigen (CEA) and non-specific crossreacting antigen (NCA).. FEBS Lett.

[OCR_01408] Lindemann F., Schlimok G., Dirschedl P., Witte J., Riethmüller G. (1992). Prognostic significance of micrometastatic tumour cells in bone marrow of colorectal cancer patients.. Lancet.

[OCR_01413] Lo Gerfo P., Herter F. P., Bennett S. J. (1972). Absence of circulating antibodies to carcinoembryonic antigen in patients with gastrointestinal malignancies.. Int J Cancer.

[OCR_01416] MacSween J. M. (1975). The antigenicity of carcinoembryonic antigen in man.. Int J Cancer.

[OCR_01431] Mavligit G. M., Ambus U., Gutterman J. U., Hersh E. M., McBride C. M. (1973). Antigen solubilized from human solid tumours: lymphocyte stimulation and cutaneous delayed hypersensitivity.. Nat New Biol.

[OCR_01424] Mavligit G. M., Gutterman J. U., McBride C. M., Hersh E. M. (1973). Cell-mediated immunity to human solid tumors: in vitro detection by lymphocyte blastogenic responses to cell-associated and solubilized tumor antigens.. Natl Cancer Inst Monogr.

[OCR_01420] Mavligit G. M., Stuckey S. (1983). Colorectal carcinoma. Evidence for circulating CEA-anti-CEA complexes.. Cancer.

[OCR_01436] Mayforth R. D., Quintáns J. (1990). Designer and catalytic antibodies.. N Engl J Med.

[OCR_01440] Miller J. F., Morahan G. (1992). Peripheral T cell tolerance.. Annu Rev Immunol.

[OCR_01444] Mittelman A., Chen Z. J., Yang H., Wong G. Y., Ferrone S. (1992). Human high molecular weight melanoma-associated antigen (HMW-MAA) mimicry by mouse anti-idiotypic monoclonal antibody MK2-23: induction of humoral anti-HMW-MAA immunity and prolongation of survival in patients with stage IV melanoma.. Proc Natl Acad Sci U S A.

[OCR_01452] Miyatake S., Handa H., Yamashita J., Yamasaki T., Ueda M., Namba Y., Hanaoka M. (1986). Induction of human glioma-specific cytotoxic T-lymphocyte lines by autologous tumor stimulation and interleukin 2.. J Neurooncol.

[OCR_01457] Monson J. R., Ramsden C., Guillou P. J. (1986). Decreased interleukin-2 production in patients with gastrointestinal cancer.. Br J Surg.

[OCR_01460] Morrissey P. J., Bressler L., Park L. S., Alpert A., Gillis S. (1987). Granulocyte-macrophage colony-stimulating factor augments the primary antibody response by enhancing the function of antigen-presenting cells.. J Immunol.

[OCR_01467] Morton B. A., O'Connor-Tressel M., Beatty B. G., Shively J. E., Beatty J. D. (1988). Artifactual CEA elevation due to human anti-mouse antibodies.. Arch Surg.

[OCR_01472] Moss B., Flexner C. (1987). Vaccinia virus expression vectors.. Annu Rev Immunol.

[OCR_01476] Moss B., Smith G. L., Gerin J. L., Purcell R. H. (1984). Live recombinant vaccinia virus protects chimpanzees against hepatitis B.. Nature.

[OCR_01480] Muraro R., Wunderlich D., Thor A., Lundy J., Noguchi P., Cunningham R., Schlom J. (1985). Definition by monoclonal antibodies of a repertoire of epitopes on carcinoembryonic antigen differentially expressed in human colon carcinomas versus normal adult tissues.. Cancer Res.

[OCR_01485] Nap M., Mollgard K., Burtin P., Fleuren G. J. (1988). Immunohistochemistry of carcino-embryonic antigen in the embryo, fetus and adult.. Tumour Biol.

[OCR_01488] Nieroda C. A., Milenic D. E., Carrasquillo J. A., Scholm J., Greiner J. W. (1995). Improved tumor radioimmunodetection using a single-chain Fv and gamma-interferon: potential clinical applications for radioimmunoguided surgery and gamma scanning.. Cancer Res.

[OCR_01495] Nijman H. W., Houbiers J. G., Vierboom M. P., van der Burg S. H., Drijfhout J. W., D'Amaro J., Kenemans P., Melief C. J., Kast W. M. (1993). Identification of peptide sequences that potentially trigger HLA-A2.1-restricted cytotoxic T lymphocytes.. Eur J Immunol.

[OCR_01501] Nossal G. J. (1983). Cellular mechanisms of immunologic tolerance.. Annu Rev Immunol.

[OCR_01505] Nossal G. J., Pike B. L. (1980). Clonal anergy: persistence in tolerant mice of antigen-binding B lymphocytes incapable of responding to antigen or mitogen.. Proc Natl Acad Sci U S A.

[OCR_01510] Oosterwijk E., Warnaar S. O., Zwartendijk J., van der Velde E. A., Fleuren G. J., Cornelisse C. J. (1988). Relationship between DNA ploidy, antigen expression and survival in renal cell carcinoma.. Int J Cancer.

[OCR_01515] Osborne M. P., Wong G. Y., Asina S., Old L. J., Cote R. J., Rosen P. P. (1991). Sensitivity of immunocytochemical detection of breast cancer cells in human bone marrow.. Cancer Res.

[OCR_01301] Peoples G. E., Goedegebuure P. S., Andrews J. V., Schoof D. D., Eberlein T. J. (1993). HLA-A2 presents shared tumor-associated antigens derived from endogenous proteins in ovarian cancer.. J Immunol.

[OCR_01520] Pertmer T. M., Eisenbraun M. D., McCabe D., Prayaga S. K., Fuller D. H., Haynes J. R. (1995). Gene gun-based nucleic acid immunization: elicitation of humoral and cytotoxic T lymphocyte responses following epidermal delivery of nanogram quantities of DNA.. Vaccine.

[OCR_01526] Pressman D., Chu T. M., Grossberg A. L. (1980). Carcinoembryonic antigen-binding immunoglobulin isolated from normal human serum by affinity chromatography.. Transplant Proc.

[OCR_01531] Ragnhammar P., Fagerberg J., Frödin J. E., Hjelm A. L., Lindemalm C., Magnusson I., Masucci G., Mellstedt H. (1993). Effect of monoclonal antibody 17-1A and GM-CSF in patients with advanced colorectal carcinoma--long-lasting, complete remissions can be induced.. Int J Cancer.

[OCR_01537] Ragnhammar P., Fagerberg J., Frödin J. E., Wersäll P., Hansson L. O., Mellstedt H. (1995). Granulocyte/macrophage-colony-stimulating factor augments the induction of antibodies, especially anti-idiotypic antibodies, to therapeutic monoclonal antibodies.. Cancer Immunol Immunother.

[OCR_01545] Raychaudhuri S., Kang C. Y., Kaveri S. V., Kieber-Emmons T., Köhler H. (1990). Tumor idiotype vaccines. VII. Analysis and correlation of structural, idiotypic, and biologic properties of protective and nonprotective Ab2.. J Immunol.

[OCR_01551] Richman P. I., Bodmer W. F. (1987). Monoclonal antibodies to human colorectal epithelium: markers for differentiation and tumour characterization.. Int J Cancer.

[OCR_01556] Riethmüller G., Johnson J. P. (1992). Monoclonal antibodies in the detection and therapy of micrometastatic epithelial cancers.. Curr Opin Immunol.

[OCR_01561] Riethmüller G., Schneider-Gädicke E., Schlimok G., Schmiegel W., Raab R., Höffken K., Gruber R., Pichlmaier H., Hirche H., Pichlmayr R. (1994). Randomised trial of monoclonal antibody for adjuvant therapy of resected Dukes' C colorectal carcinoma. German Cancer Aid 17-1A Study Group.. Lancet.

[OCR_01567] Robins R. A., Denton G. W., Hardcastle J. D., Austin E. B., Baldwin R. W., Durrant L. G. (1991). Antitumor immune response and interleukin 2 production induced in colorectal cancer patients by immunization with human monoclonal anti-idiotypic antibody.. Cancer Res.

[OCR_01579] Rodenburg C. J., Cornelisse C. J., Heintz P. A., Hermans J., Fleuren G. J. (1987). Tumor ploidy as a major prognostic factor in advanced ovarian cancer.. Cancer.

[OCR_01584] Rosa F. M., Fellous M. (1988). Regulation of HLA-DR gene by IFN-gamma. Transcriptional and post-transcriptional control.. J Immunol.

[OCR_01587] Rosenberg S. A., Lotze M. T., Yang J. C., Aebersold P. M., Linehan W. M., Seipp C. A., White D. E. (1989). Experience with the use of high-dose interleukin-2 in the treatment of 652 cancer patients.. Ann Surg.

[OCR_01592] Schlimok G., Funke I., Bock B., Schweiberer B., Witte J., Riethmüller G. (1990). Epithelial tumor cells in bone marrow of patients with colorectal cancer: immunocytochemical detection, phenotypic characterization, and prognostic significance.. J Clin Oncol.

[OCR_01598] Shortman K. (1992). Cellular aspects of early T-cell development.. Curr Opin Immunol.

[OCR_01602] Sidransky D., Tokino T., Hamilton S. R., Kinzler K. W., Levin B., Frost P., Vogelstein B. (1992). Identification of ras oncogene mutations in the stool of patients with curable colorectal tumors.. Science.

[OCR_01607] Sieff C. A., Emerson S. G., Donahue R. E., Nathan D. G., Wang E. A., Wong G. G., Clark S. C. (1985). Human recombinant granulocyte-macrophage colony-stimulating factor: a multilineage hematopoietin.. Science.

[OCR_01612] Slingluff C. L., Cox A. L., Henderson R. A., Hunt D. F., Engelhard V. H. (1993). Recognition of human melanoma cells by HLA-A2.1-restricted cytotoxic T lymphocytes is mediated by at least six shared peptide epitopes.. J Immunol.

[OCR_01618] Slovin S. F., Lackman R. D., Ferrone S., Kiely P. E., Mastrangelo M. J. (1986). Cellular immune response to human sarcomas: cytotoxic T cell clones reactive with autologous sarcomas. I. Development, phenotype, and specificity.. J Immunol.

[OCR_01623] Sorokin J. J., Kupchik H. Z., Zamcheck N. (1973). Brief communication: carcinoembryonic antigen in colon cancer: absence in perchloric acid precipitates of plasma.. J Natl Cancer Inst.

[OCR_01628] Staab H. J., Anderer F. A., Stumpf E., Fischer R. (1980). Are circulating CEA immune complexes a prognostic marker in patients with carcinoma of the gastrointestinal tract?. Br J Cancer.

[OCR_01644] Steplewski Z., Herlyn D., Lubeck M., Kimoto Y., Herlyn M., Koprowski H. (1986). Mechanisms of tumor growth inhibition.. Hybridoma.

[OCR_01633] Steplewski Z., Lubeck M. D., Koprowski H. (1983). Human macrophages armed with murine immunoglobulin G2a antibodies to tumors destroy human cancer cells.. Science.

[OCR_01651] Thompson J. A., Grunert F., Zimmermann W. (1991). Carcinoembryonic antigen gene family: molecular biology and clinical perspectives.. J Clin Lab Anal.

[OCR_01647] Thompson J., Zimmermann W. (1988). The carcinoembryonic antigen gene family: structure, expression and evolution.. Tumour Biol.

[OCR_01656] Trauth B. C., Klas C., Peters A. M., Matzku S., Möller P., Falk W., Debatin K. M., Krammer P. H. (1989). Monoclonal antibody-mediated tumor regression by induction of apoptosis.. Science.

[OCR_01661] Tsang K. Y., Zaremba S., Nieroda C. A., Zhu M. Z., Hamilton J. M., Schlom J. (1995). Generation of human cytotoxic T cells specific for human carcinoembryonic antigen epitopes from patients immunized with recombinant vaccinia-CEA vaccine.. J Natl Cancer Inst.

[OCR_01667] Tynan K., Olsen A., Trask B., de Jong P., Thompson J., Zimmermann W., Carrano A., Mohrenweiser H. (1992). Assembly and analysis of cosmid contigs in the CEA-gene family region of human chromosome 19.. Nucleic Acids Res.

[OCR_01673] Ura Y., Ochi Y., Hamazu M., Ishida M., Nakajima K., Watanabe T. (1985). Studies on circulating antibody against carcinoembryonic antigen (CEA) and CEA-like antigen in cancer patients.. Cancer Lett.

[OCR_01678] Wadhwa M., Bird C., Fagerberg J., Gaines-Das R., Ragnhammar P., Mellstedt H., Thorpe R. (1996). Production of neutralizing granulocyte-macrophage colony-stimulating factor (GM-CSF) antibodies in carcinoma patients following GM-CSF combination therapy.. Clin Exp Immunol.

[OCR_01684] Wang B., Boyer J., Srikantan V., Coney L., Carrano R., Phan C., Merva M., Dang K., Agadjanan M., Gilbert L. (1993). DNA inoculation induces neutralizing immune responses against human immunodeficiency virus type 1 in mice and nonhuman primates.. DNA Cell Biol.

[OCR_01691] Williams A. F., Barclay A. N. (1988). The immunoglobulin superfamily--domains for cell surface recognition.. Annu Rev Immunol.

[OCR_01695] Winter G., Milstein C. (1991). Man-made antibodies.. Nature.

[OCR_01709] Wolff J. A., Ludtke J. J., Acsadi G., Williams P., Jani A. (1992). Long-term persistence of plasmid DNA and foreign gene expression in mouse muscle.. Hum Mol Genet.

[OCR_01704] Wolff J. A., Malone R. W., Williams P., Chong W., Acsadi G., Jani A., Felgner P. L. (1990). Direct gene transfer into mouse muscle in vivo.. Science.

[OCR_01714] Wyllie A. H., Kerr J. F., Currie A. R. (1980). Cell death: the significance of apoptosis.. Int Rev Cytol.

[OCR_01697] Wölfel T., Schneider J., Meyer Zum Büschenfelde K. H., Rammensee H. G., Rötzschke O., Falk K. (1994). Isolation of naturally processed peptides recognized by cytolytic T lymphocytes (CTL) on human melanoma cells in association with HLA-A2.1.. Int J Cancer.

[OCR_01718] Yang N. S., Burkholder J., Roberts B., Martinell B., McCabe D. (1990). In vivo and in vitro gene transfer to mammalian somatic cells by particle bombardment.. Proc Natl Acad Sci U S A.

[OCR_01723] Zhou X., Berglund P., Zhao H., Liljeström P., Jondal M. (1995). Generation of cytotoxic and humoral immune responses by nonreplicative recombinant Semliki Forest virus.. Proc Natl Acad Sci U S A.

